# Evolthon: A community endeavor to evolve lab evolution

**DOI:** 10.1371/journal.pbio.3000182

**Published:** 2019-03-29

**Authors:** Sivan Kaminski Strauss, Dvir Schirman, Ghil Jona, Aaron N. Brooks, Aditya M. Kunjapur, Alex N. Nguyen Ba, Alice Flint, Andras Solt, Andreas Mershin, Atray Dixit, Avihu H. Yona, Bálint Csörgő, Bede Phillip Busby, Bianca P. Hennig, Csaba Pál, Daniel Schraivogel, Daniel Schultz, David G. Wernick, Deepa Agashe, Dikla Levi, Dmitry Zabezhinsky, Dor Russ, Ehud Sass, Einat Tamar, Elad Herz, Emmanuel D. Levy, George M. Church, Idan Yelin, Iftach Nachman, Jeffrey E. Gerst, Joseph M. Georgeson, Katarzyna P. Adamala, Lars M. Steinmetz, Marc Rübsam, Markus Ralser, Michael Klutstein, Michael M. Desai, Nilima Walunjkar, Ning Yin, Noa Aharon Hefetz, Noah Jakimo, Olga Snitser, Omri Adini, Prashant Kumar, Rachel Soo Hoo Smith, Razi Zeidan, Ronen Hazan, Roni Rak, Roy Kishony, Shannon Johnson, Shira Nouriel, Sibylle C. Vonesch, Simmie Foster, Tal Dagan, Tanita Wein, Thrasyvoulos Karydis, Timothy M. Wannier, Timothy Stiles, Viridiana Olin-Sandoval, William F. Mueller, Yinon M. Bar-On, Orna Dahan, Yitzhak Pilpel

**Affiliations:** 1 Department of Molecular Genetics, Weizmann Institute of Science, Rehovot, Israel; 2 Department of Life Sciences Core Facilities, Weizmann Institute of Science, Rehovot, Israel; 3 European Molecular Biology Laboratory (EMBL), Genome Biology Unit, Heidelberg, Germany; 4 Department of Genetics, Harvard Medical School, Boston, Massachusetts, United States of America; 5 Department of Organismic and Evolutionary Biology, Harvard University, Cambridge, Massachusetts, United States of America; 6 Department of Biochemistry, University of Cambridge, Cambridge, United Kingdom; 7 Massachusetts Institute of Technology, Center for Bits and Atoms, Cambridge, Massachusetts, United States of America; 8 Broad Institute of MIT and Harvard, Cambridge, Massachusetts, United States of America; 9 Harvard-MIT Division of Health Sciences and Technology, Cambridge, Massachusetts, United States of America; 10 Physics of Living Systems, Department of Physics, Massachusetts Institute of Technology, Cambridge, Massachusetts, United States of America; 11 Institute of Biochemistry, Biological Research Centre of the Hungarian Academy of Sciences, Szeged, Hungary; 12 European Molecular Biology Laboratory, European Bioinformatics Institute (EMBL-EBI), Wellcome Genome Campus, Cambridge, United Kingdom; 13 Department of Microbiology and Immunology, Geisel School of Medicine at Dartmouth, Hanover, New Hampshire, United States of America; 14 Department of Plant and Environmental Sciences, Weizmann Institute of Science, Rehovot, Israel; 15 National Centre for Biological Sciences, Bangalore, India; 16 Faculty of Biology, Technion–Israel Institute of Technology, Haifa, Israel; 17 Department of Structural Biology, Weizmann Institute of Science, Rehovot, Israel; 18 Department of Biochemistry and Molecular Biology, George S. Wise Faculty of Life Sciences, Tel Aviv University, Tel Aviv, Israel; 19 University of Minnesota, Minneapolis, Minnesota, United States of America; 20 Stanford Genome Technology Center, Stanford University, Palo Alto, California, United States of America; 21 Department of Genetics, Stanford University School of Medicine, Stanford, California, United States of America; 22 The Molecular Biology of Metabolism laboratory, The Francis Crick Institute, London, United Kingdom; 23 Department of Biochemistry, Charitè University Medicine, Berlin, Germany; 24 Faculty of Dental Medicine, The Hebrew University of Jerusalem, Jerusalem, Israel; 25 Department of Physics, Harvard University, Cambridge, Massachusetts, United States of America; 26 Faculty of Computer Science, Technion–Israel Institute of Technology, Haifa, Israel; 27 Harvard University Extension School, Cambridge, Massachusetts, United States of America; 28 Harvard Medical School, Boston, Massachusetts, United States of America; 29 Massachusetts General Hospital, Boston, Massachusetts, United States of America; 30 Boston Children’s Hospital, Boston, Massachusetts, United States of America; 31 Institute of Microbiology, Kiel University, Kiel, Germany; 32 BosLab, Somerville, Massachusetts, United States of America; 33 Department of Nutrition Physiology, Instituto Nacional de Ciencias Medicas y Nutricion Salvador Zubiran, Mexico City, Mexico; University of Bath, UNITED KINGDOM

## Abstract

In experimental evolution, scientists evolve organisms in the lab, typically by challenging them to new environmental conditions. How best to evolve a desired trait? Should the challenge be applied abruptly, gradually, periodically, sporadically? Should one apply chemical mutagenesis, and do strains with high innate mutation rate evolve faster? What are ideal population sizes of evolving populations? There are endless strategies, beyond those that can be exposed by individual labs. We therefore arranged a community challenge, Evolthon, in which students and scientists from different labs were asked to evolve *Escherichia coli* or *Saccharomyces cerevisiae* for an abiotic stress—low temperature. About 30 participants from around the world explored diverse environmental and genetic regimes of evolution. After a period of evolution in each lab, all strains of each species were competed with one another. In yeast, the most successful strategies were those that used mating, underscoring the importance of sex in evolution. In bacteria, the fittest strain used a strategy based on exploration of different mutation rates. Different strategies displayed variable levels of performance and stability across additional challenges and conditions. This study therefore uncovers principles of effective experimental evolutionary regimens and might prove useful also for biotechnological developments of new strains and for understanding natural strategies in evolutionary arms races between species. Evolthon constitutes a model for community-based scientific exploration that encourages creativity and cooperation.

## Abstract 2

This Community Page article describes Evolthon; a first-of-its-kind community-based effort, involving about 30 participant labs around the world, aiming to explore the best strategy for evolving microorganisms to cope with an environmental challenge.

## Introduction

The known saying, “Nothing in biology makes sense except in light of evolution,” [1] clearly exemplifies the pivotal role of evolutionary thinking in biology. Classical investigations in evolution are based on observing and comparing organisms in nature, and they require inference of the past conditions and species history. Though extremely insightful, this approach can be effectively complemented by “lab-evolution,” a research paradigm in which organisms, typically microbes, are evolved in the lab. In this controlled setup, species can be challenged by changing environmental conditions, e.g., starvation, exposure to antibiotic drugs, high temperature, high salinity [2–5], or by perturbing their genes [6–8], and then they can be followed as they evolve, inspecting a diversity of physiological and genomic means of adaptation. Therefore, rather than simply observing a snapshot, an entire evolutionary “movie” can be followed, during which the environment is not only known but can also be controlled and manipulated. The Long Term Evolutionary Experiment [2] is a famous experiment that essentially established the field, and in recent years many experiments followed [9–12].

Consider then the following challenge: you are given a microbe and you are asked to evolve it in the lab towards a new challenge, say to extreme temperature or to a toxic drug. What evolutionary regime will achieve “best” results? Naturally, one would expose the population to the challenge (e.g., high temperature or the drug), but open questions would include: (i) What is the optimal level of exposure to the stress? (ii) Should the stress level be constant throughout the experiment, or should it increase, decrease, oscillate, or fluctuate randomly with time? (iii) What should be the population size? Small populations feature evolutionary bottleneck and high effect of drift; (iv) If the organism can exercise sexual mating, should that be allowed? (v) Should mutation rate be manipulated, e.g., by exposing the evolving cells to a mutagen, or by working with a strain that features high mutation rate? (vi) Should cells be allowed to cycle between all stages of growth, as in serial dilution regimes[2], or should they be grown in a chemostat in constant logarithmic phase [13]?

One can be very creative in designing an evolutionary experiment, and the number of degrees of freedom is essentially unlimited. Post factum, one could ask, how did the evolutionary strategy employed affected performance? For example, it has been shown in yeast that exposure to an abruptly applied challenge, high temperature, as opposed to incremental increase in the temperature, pushed cells to evolve very different solutions. When exposed to an abrupt increase in temperature, yeast evolved through aneuploidy, a solution that proves to be maladaptive in other stresses, and that might not endure well after short relaxation periods [3]. Therefore, an interesting possibility is that the adaptation regime applied during evolution would affect the stability and generality of the adaptation.

Although many works were done looking at individual evolution strategies, there is no larger-scale study aiming to compare the effects of different evolution strategies. In a first of its kind initiative in the evolution biology community, we, along with various experimental-evolution groups worldwide, have participated in the first Evolthon Challenge, a tournament that challenged participants to come up with creative ways to evolve microorganisms in the lab. We focused here on either *Escherichia coli* or *Saccharomyces cerevisiae*, and the challenge was adaptation to low temperature. The inspiration for the Evolthon Challenge came from the successes of other community efforts to advance and generate new thought in other fields, most notably, Axelrod's Tournament in evolutionary game theory [14], the systems biology competition Dialogue for Reverse Engineering Assessments and Methods (DREAM)[15], and the International Genetically Engineered Machine (iGEM) competition in the field of synthetic biology [16]. We were eager to create a platform to enable the joint exploration of the range of possibilities in evolving a trait with the belief that such an endeavor will allow researchers and students to explore, be creative, collaborate, share knowledge and insight, to educate themselves through this process, and contribute knowledge and advance the field of lab evolution. The ultimate goal is to seed a collection of creative lab evolution strategies and generate a first-of-its-kind lab evolution strategies database, that will grow further past this initial publication, from which researchers and biotechnologists will be able to select and adapt further.

## The Evolthon Challenge outline

In the Evolthon Challenge, participants faced a clearly defined challenge: evolve a microbe, either *E*. *coli* or *S*. *cerevisiae*, so that it will be as fit as possible at lower than optimal temperature, i.e., 20°C or 15°C, respectively. Each participant received from the organizers a uniquely genome-barcoded strain (either bacteria or yeast) and was given three months to evolve or engineer the strain toward the set challenge in whatever method they wanted before sending it back to the organizers’ lab. The strains were then competed with one another to determine their fitness under the low temperature challenge and on other challenges that were not announced originally. Participants were not allowed to introduce “bio-weapons” to directly toxify other competing strains, but other than that, they were allowed to use genetics, genome engineering, chemical treatments, exposure to environmental stress, etc. (The full set of instructions can be found in S1 Text).

The fitness of each strain was assessed in the organizers’ lab in two standardized ways; first, each strain was grown in isolation, its growth curve under low temperature was generated and used to deduce various growth parameters that were compared to the ancestor’s values. The second means for evaluation was a pooled competition experiment, in which all strains, from each of the two species separately, were grown together and were allowed to compete, thus revealing the relative fitness of the strains to one another. Sequencing the barcodes during the competition enabled the determination of the relative frequency of each strain across a time course, thus determining its relative fitness. To account for biases originating from lab-to-lab technical variation, the competition between all evolving strains was carried out twice, once in the organizers’ lab and once in an additional lab.

Evolution is an arena of trade-offs. For example, a strain can become well adapted to one extreme condition, but it might lose its fitness to the original environment, featuring various levels of “evolutionary memory.” Furthermore, it is possible that by adapting to one stress the evolved strain may have “generalized,” i.e., it might now become adapted to other stresses as well. A particularly interesting notion is that the evolutionary strategy used to evolve the strain for the original challenge could affect such tradeoffs and ability to “memorize” the adaptation to the original temperature, or to “generalize” and become fit in other conditions too. We therefore also assessed the strains’ ability to cope with other growth conditions whose nature were not announced to the participants ahead of time.

## Results

**Fig 1:**
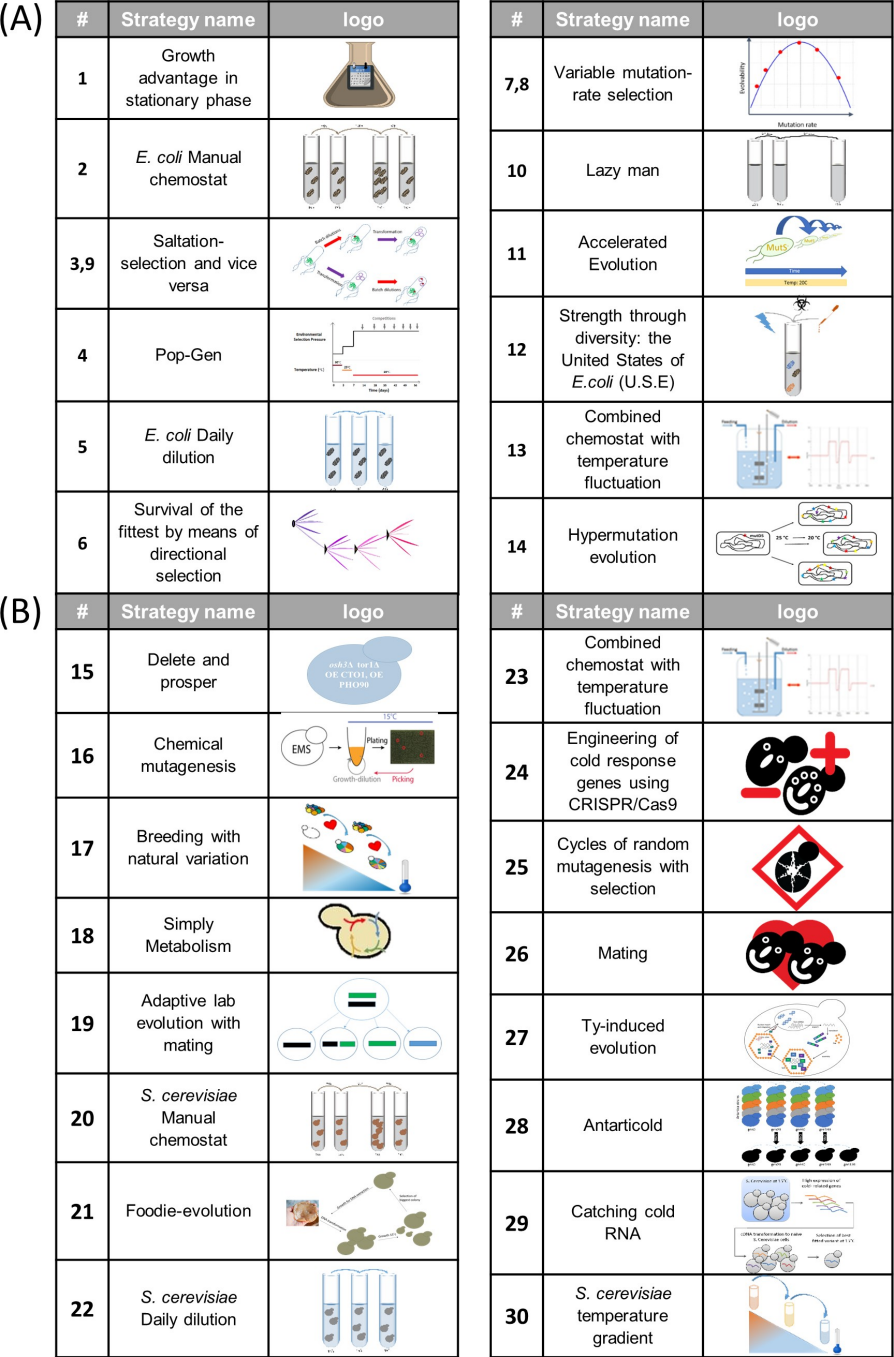
Summary of the strategies employed in Evolthon. All strategies used in Evolthon are listed each strategy is characterize by identifying number, name, and logo. (A) Strategies used *for E*.*coli*. (B) Strategies used for *S*. *cerevisiae*. Additional details of each strategy can be found in S1 Table and S2 Text. Pop-Gen, Population Genetics; CRISPR, clustered regularly interspaced short palindromic repeats.

The Evolthon Challenge announcement generated great interest among the lab evolution community, with more than 20 labs from 6 countries and 12 academic institutes opting to participate. Each participant designed its own strategy for the evolution of its strain. The different strategies that participants used were very diverse, and they included strategies based on increasing mutagenesis, genomic engineering, mating (in yeast), and more. Fig 1 summarizes the strategies employed.

**Fig 2:**
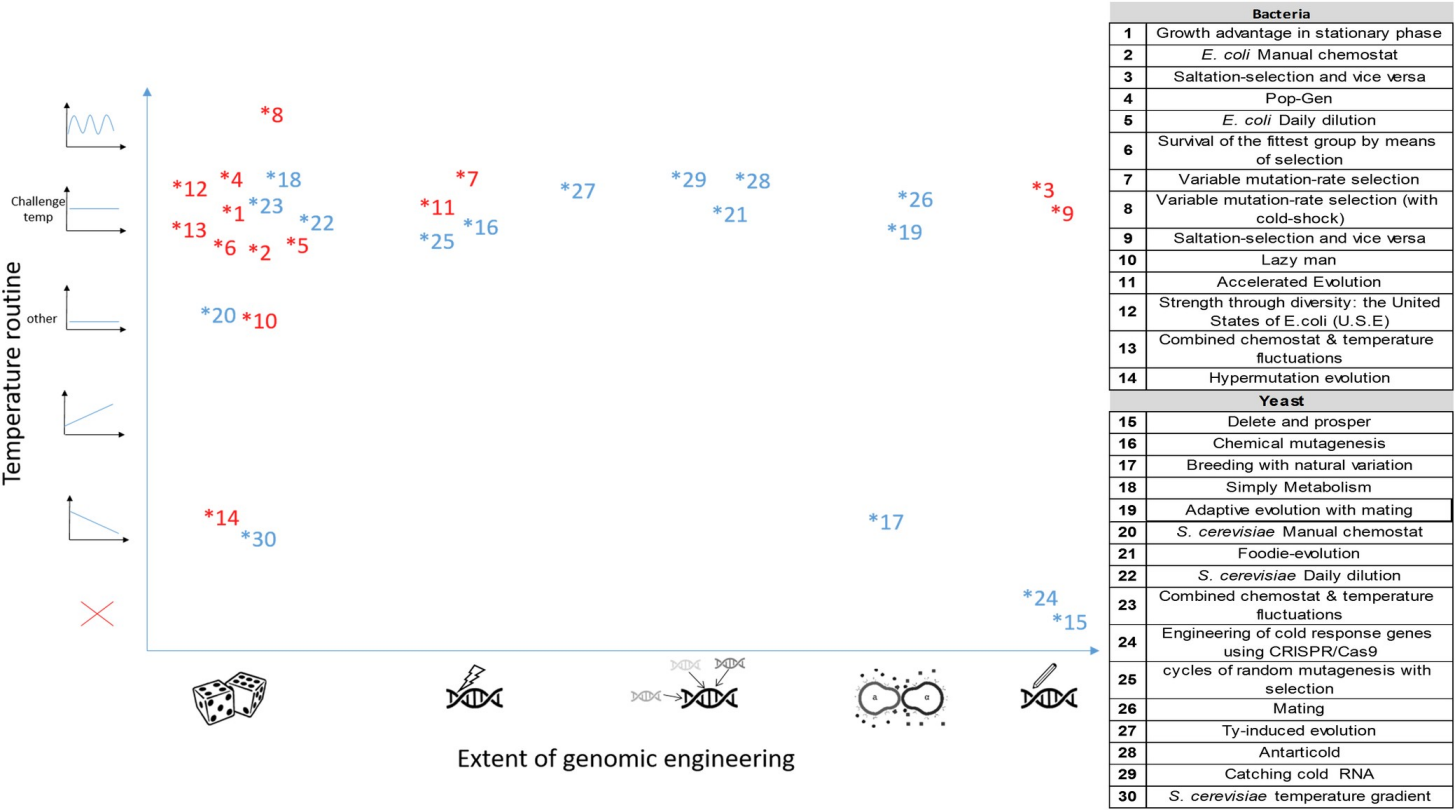
Schematic illustration of the different evolutionary strategies location in the conceptual plan. We conceptually and qualitatively projected all 30 evolutionary strategies onto a plane that is spanned by two principled characteristics of many of the strategies. The x axis denotes the extent of genome engineering and mutagenesis used. The left most strategies used no engineering, the second used mutagenesis, the third used DNA transformation, the fourth used mating (in yeast), and the right most used genome engineering. The y axis denotes the temperature versus evolutionary time regimen experienced by cells during evolution, with strategies exposing cells to fluctuating temperature, constant temperature, monotonically increasing or decreasing temperature, and a strategy (marked by a red X) that involved engineering with no lab evolution. Colors represent organism, *E*. *coli* (blue) or *S*. *cerevisiae* (red).

Fig 2 localizes the various strategies on a conceptual plane that is spanned by two “principal axes;” the horizontal axis characterizes the extent of genetic manipulation, and the vertical one characterizes the environment regime employed. Strategies on the far right side employed elaborate genome engineering; those on the left side did not intervene genetically at all with the evolving genomes, whereas the middle of this axis represents strategies that were based on enhanced mutagenesis, sexual mating with other diverse strains, random integration of DNA fragments from various sources, etc. For example, some participants engineered genomes to introduce beneficial mutations at various levels of design, ranging from knock-in of genes induced as cold-resistance proteins, to random transformation of genomic DNA from cold-resistant yeast strains. A cluster of strategies in yeast used sexual mating. The vertical axis represents the environmental exposure regime, when applicable, exercised in each strategy. High on the vertical axis are strategies that exposed cells to fluctuating temperature, and lower on this axis are those kept at a constant temperature. Some participants evolved their strains under a constant temperature, either the announced low temperature, or either constantly higher or lower temperatures throughout the evolution. Lowest on the y axis are strategies that monotonically change the temperature during evolution and those in which no evolution was employ. A noteworthy strategy is the daily-dilution routine performed at the desired condition (performed in a “Lenski-style” lab evolution [2]), because it is the common strategy used in lab evolution experiments. It was implemented both in *E*. *coli* and in *S*. *cerevisiae*, respectively, at 20°C or 15°C (strategies #5 “*E*. *coli* Daily dilution” and #22 “*S*. *cerevisiae* Daily dilution”). Strategies also differed in other aspects of their lab evolution protocol, such as number of generation, population size, and the environmental settings. More information about each of the strategies can be found in S2 Text and S1 Table.

The spread of exercised strategies on this plane also exposes regions that were not explored here. For example, none of the participants combined rational design with evolution under changing temperatures. Furthermore, although some strategies decreased the temperature gradually along evolution, no strategy featured a gradual increase of temperature. Such evolutionary regimes could be examined in future.

## Most evolved strains exhibited improved fitness in the cold environment

**Fig 3:**
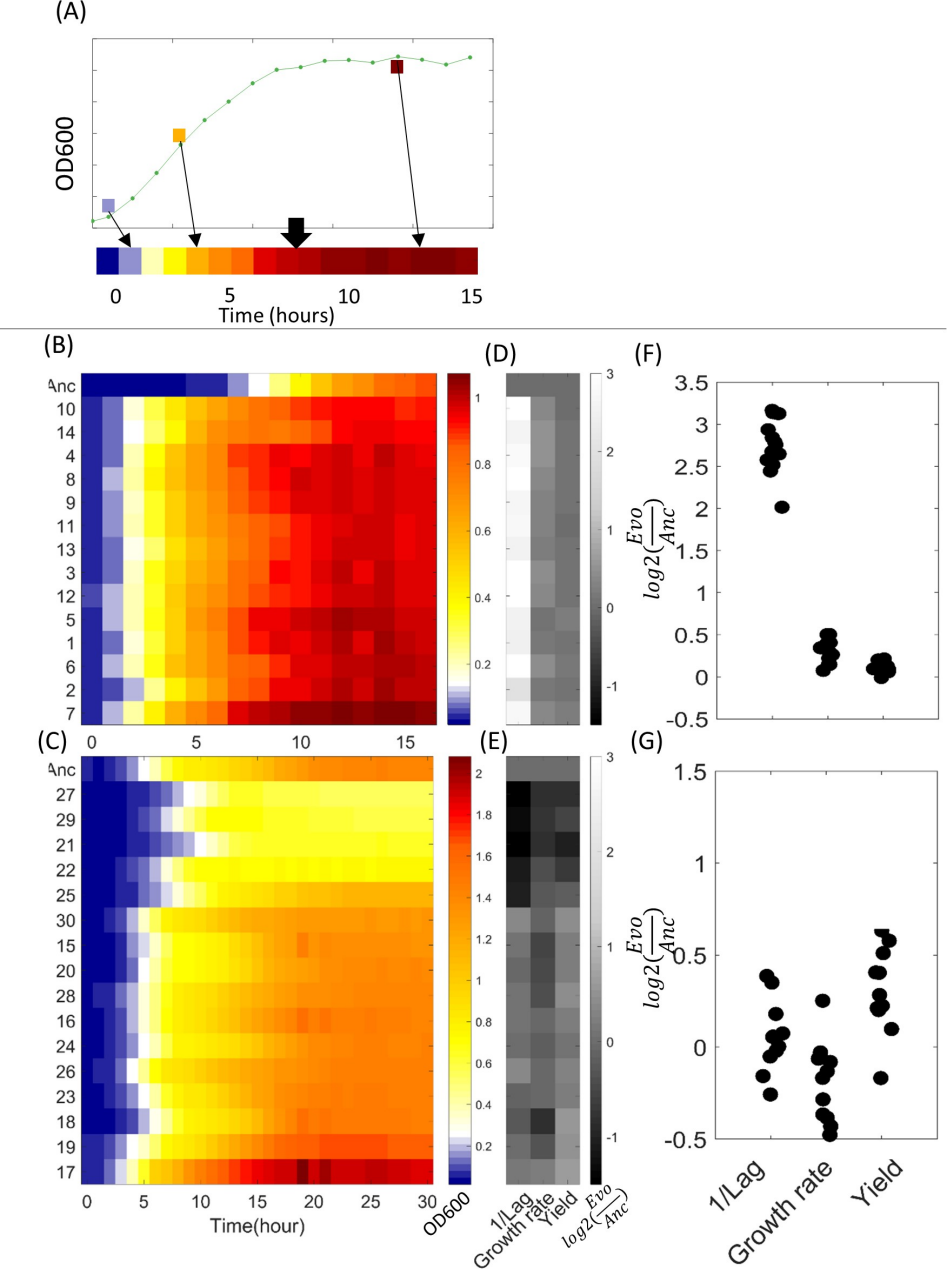
Growth experiments of individual strains. All strains were grown for approximatley 30 hours in 15°C and 20°C (*E*. *coli* and *S*. *cerevisiae*, respectively), while measuring OD600 every approximately 1.5 hours. (A) Schematic representation of transforming growth curves into heat map figure. Each point in the growth curve is colored based on its OD600 value to obtain the heat map figure. (B-C) Growth in heat map format. Each row corresponds to a strain. Color bar represents OD600 values. Growth experiments were done in 11 replicates per strain. (B) *E*. *coli* (SD doesn’t exceed 0.02). (C) *S*. *cerevisiae* (SD doesn’t exceed 0.17). Strains in each species are sorted in ascending order according to final OD. (D-G) Growth parameters (lag, growth rate, and yield) were calculated based on a mathematical model for growth (for details, see S3 Text). Color bar represents log2(Evol/Anc) for each growth parameter. (D, F) *E*. *coli*; (E, G) *S*. *cerevisiae*. Strains key: *E*. *coli strains*: (1) Growth advantage in stationary phase, (2) *E*. *coli* Manual chemostat, (3) Saltation-selection and vice versa, (4) Pop-Gen, (5) *E*. *coli* daily dilution, (6) Survival of the fittest group by means of selection, (7) Variable mutation-rate selection, (8) Variable mutation-rate selection (with cold shock), (9) Saltation selection and vice versa, (10) Lazy man, (11) Accelerated Evolution, (12) Strength through diversity: the United States of *E*.*coli* (U.S.E), (13) Combined chemostat and temperature fluctuations, (14) Hypermutation evolution. *S*. *cerevisiae strains*: (15) Delete and prosper, (16) Chemical mutagenesis, (17) Breeding with natural variation, (18) Simply Metabolism, (19) Adaptive evolution with mating, (20) *S*. *cerevisiae* Manual chemostat, (21) Foodie-evolution, (22) *S*. *cerevisiae* Daily dilution, (23) Combined chemostat and temperature fluctuations, (24) Engineering of cold response genes using CRISPR/Cas9, (25) cycles of random mutagenesis with selection, (26) Mating, (27) Ty-induced evolution, (28) Antarticold, (29) Catching cold RNA, (30) *S*. *cerevisiae* temperature gradient. Raw data and quantification data for this figure can be found in S1 Data.

Comparison of the fitness of the ancestral strains with those of the evolved strains, measured by individual growth curves, revealed that all evolved *E*. *coli* strains significantly improved their fitness in the cold environment as compared to their ancestor (Fig 3A and 3B). In *S*. *cerevisiae*, the picture was more complex, in which some strains improved their fitness whereas the fitness of others either did not change or actually declined (Fig 3A and 3C). To further quantify how strains adapted to the cold environment, we analyzed the individual growth curves and extracted three growth parameters for each strain: lag phase duration, growth rate in the exponential phase, and the yield (maximal optical density [OD]) at the stationary phase [17] (Table 1). As can be seen from Fig 3D and 3E, the evolved *E*. *coli* and *S*. *cerevisiae* strains (respectively) behave differently. All *E*. *coli* strains mainly evolved by significantly shortening the lag phase duration, and they also improved their growth rate to some extent, whereas their yield showed little improvement (Fig 3F). In *S*. *cerevisiae*, in addition to strains that improved their fitness, there were strains in which none of the parameters were improved, and even strains that performed worse than the ancestor, mainly due to increase of lag time (Fig 3G). Moreover, unlike *E*. *coli* that mainly improved its lag, the fittest strains in *S*. *cerevisiae* primarily improved their yield (Fig 3D and 3E). The ancestral strains of *S*. *cerevisiae* and *E*. *coli* have different growth dynamics, especially a different lag phase duration (under the low temperature regimes). In *S*. *cerevisiae*, the lag phase is approximately 11% of the entire growth cycle (5 hours of lag phase out of 45 hours until stationary). In *E*. *coli*, the lag phase duration is also 5 hours, but the entire growth cycle duration is 25 hours (thus lag phase covers some 20% of the cycle) (Fig 3B and 3C Top rows). In light of this dynamics, it seems that the benefit of shortening the lag phase is higher in *E*. *coli* than in *S*. *cerevisiae*. These results indicate that, unlike a potential naïve expectation, increase in growth rate might be less common in adapting to a new environment; in contrast shortening lag phase appears to be the immediate avenue for adaptation in *E*.*coli*. Shortening of lag phase was revealed as the main means of adaptation in *E*.*coli* population that were not exposed to such abiotic stress [18] but rather evolved to utilize nonfavorable carbon source. This commonality suggests that evolution of shortened lag phase in *E*. *coli* may be a common adaptation mean featured in different types of conditions. Because both *E*. *coli* and *S*. *cerevisiae* were evolved under the same type of stress, i.e., cold temperature, our data allowed us to compare the type of improvement featured by the two organisms. By looking on the correlation across evolving strains, in their improvements in each of the growth phases, we note a difference between the two types of organisms. Whereas in yeasts strategies that improved the performance of the cells in one phase typically improved performance in other phases, in *E*. *coli*, correlations exist only between lag phase and growth phase improvements (S1 Fig).

**Table 1:** Summary of strains’ performance. For each strategy and the ancestral strain the growth parameters (1/lag, growth rate, and yield) calculated from individual growth curves (see **S3 Text**) are shown. Fitness values are calculated using a maximum likelihood algorithm (see **S3 Text**) based on the pool competition. Fitness was only calculated for strains with more than 10 reads at the beginning of the competition (otherwise ND is assigned).

**Bacteria**					
**#**	**Strategy name**	**Growth Curves parameters**			**Fitness**
		**1/Lag**	**Growth rate**	**yield**	
**anc**	**-**	0.096	0.438	0.931	
**1**	Growth advantage in stationary phase	0.509	0.497	1.001	−0.234
**2**	*E*. *coli* Manual chemostat	0.380	0.493	1.010	−0.028
**3**	Saltation-selection and vice versa	0.733	0.579	0.994	−0.174
**4**	Pop-Gen	0.697	0.620	0.974	−0.097
**5**	*E*. *coli* Daily dilution	0.577	0.451	1.017	−0.173
**6**	Survival of the fittest group by means of selection	0.819	0.586	1.015	0.013
**7**	Variable mutation-rate selection	0.649	0.554	1.066	0.086
**8**	Variable mutation-rate selection (with cold-shock)	0.813	0.587	0.985	−0.130
**9**	Saltation-selection and vice versa	0.808	0.525	0.983	−0.159
**10**	Lazy man	0.852	0.557	0.983	−0.110
**11**	Accelerated Evolution	0.678	0.527	0.945	−0.076
**12**	Strength through diversity: the United States of *E*.*coli* (U.S.E)	0.838	0.567	0.988	−0.250
**13**	Combined chemostat and temperature fluctuations	0.565	0.495	0.992	−0.243
**14**	Hypermutation evolution	0.602	0.608	0.941	−0.097
**Yeast**					
**#**	**Strategy name**	**Growth Curves parameters**			**Fitness**
		**1/Lag**	**Growth rate**	**yield**	
**anc**	**-**	0.137	0.196	1.196	** **
**15**	Delete and prosper	0.132	0.149	1.411	−0.283
**16**	Chemical mutagenesis	0.145	0.169	1.491	−0.187
**17**	Breeding with natural variation	0.162	0.224	1.963	0.281
**18**	Simply Metabolism	0.105	0.143	1.621	−0.437
**19**	Adaptive evolution with mating	0.143	0.129	1.860	−0.762
**20**	*S*. *cerevisiae* Manual chemostat	0.143	0.143	1.353	ND
**21**	Foodie-evolution	0.040	0.090	0.639	ND
**22**	*S*. *cerevisiae* Daily dilution	0.065	0.159	0.724	ND
**23**	Combined chemostat and temperature fluctuations	0.131	0.161	1.490	−0.121
**24**	Engineering of cold response genes using CRISPR/Cas9	0.144	0.151	1.363	−0.298
**25**	cycles of random mutagenesis with selection	0.069	0.154	1.027	ND
**26**	Mating	0.178	0.219	1.319	−0.003
**27**	Ty-induced evolution	0.043	0.096	0.691	−0.128
**28**	Antarticold	0.149	0.153	1.511	−0.351
**29**	Catching cold RNA	0.054	0.129	0.749	−0.318
**30**	*S*. *cerevisiae* temperature gradient	0.149	0.195	1.391	−0.062

**Abbreviations:** anc, ancestor; ND, Not Determined.

## Evaluating fitness using the pooled competition assay

**Fig 4:**
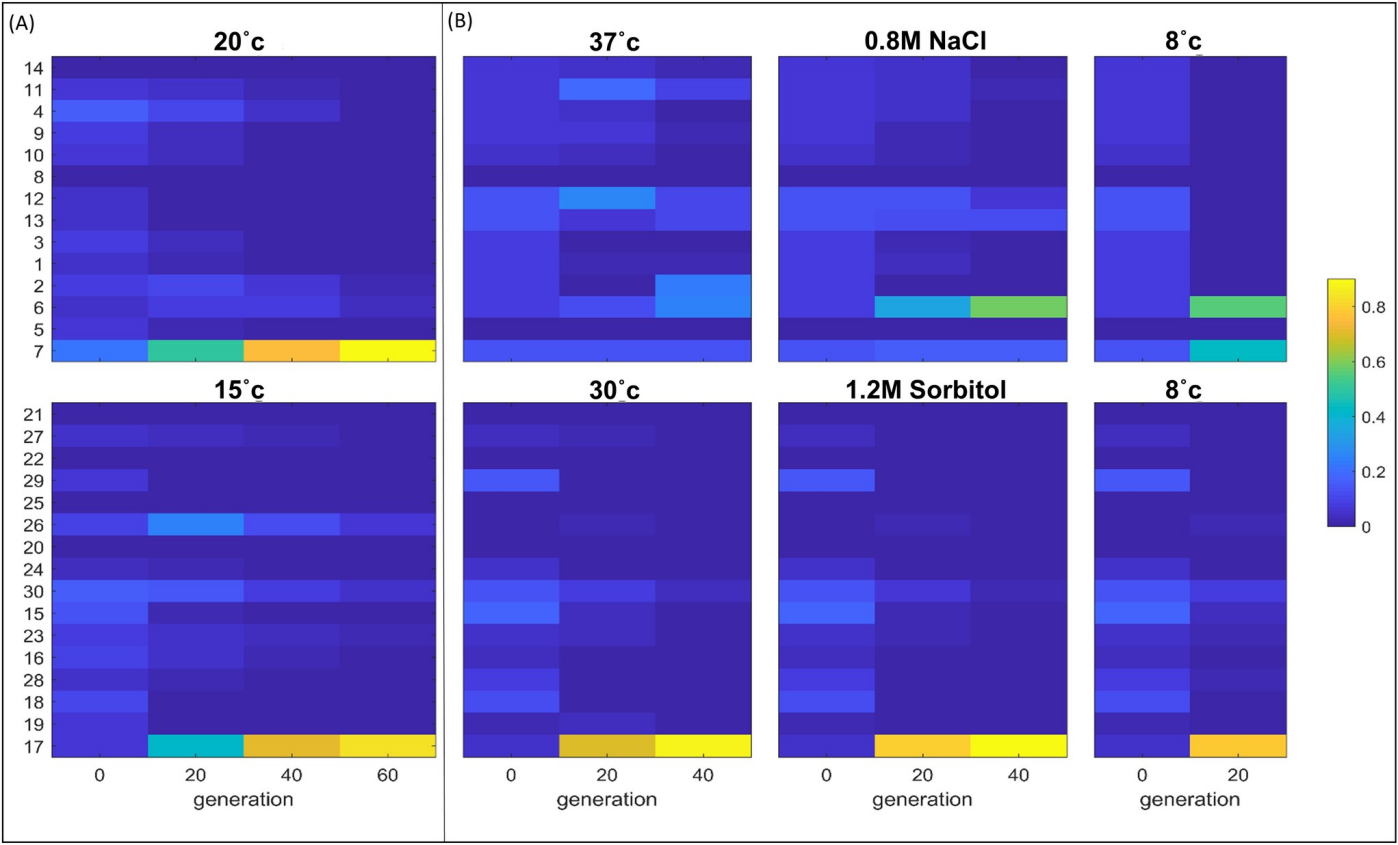
Pooled competition. Strains were mixed and grown for several dozens of generations in serial dilution regimes under different growth conditions (see S3 Text for details). At different time points during the competition, barcodes were sequenced, and their frequencies are shown. (A) Challenge conditions to which strains were evolved (15°C and 20°C for *E*. *coli* and *S*. *cerevisiae*, respectively). Color bar represents the frequency of the strains barcode reads from total number of reads. (B) Other challenges (“evolutionary memory,” 37°C and 30°C for *E*. *coli* and *S*. *cerevisiae*, respectively; “generalization,” 0.8M NaCl and 1.2M sorbitol for *E*. *coli* and *S*. *cerevisiae*; “extremity,” 8°C for both *E*. *coli* and *S*. *cerevisiae)*. Color bar represents the frequency of the strains barcode reads from total number of reads. Upper panels present *E*. *coli* competition results; lower panels present *S*. *cerevisiae* competition results. Strains key: *E*. *coli* strains: (1) Growth advantage in stationary phase, (2) *E*. *coli* Manual chemostat, (3) Saltation-selection and vice versa, (4) Pop-Gen, (5) *E*. *coli* Daily dilution, (6) Survival of the fittest group by means of selection, (7) Variable mutation-rate selection, (8) Variable mutation-rate selection (with cold-shock), (9) Saltation-selection and vice versa, (10) Lazy man, (11) Accelerated Evolution, (12) Strength through diversity: the United States of *E*.*coli* (U.S.E), (13) Combined chemostat and temperature fluctuations, (14) Hypermutation evolution. *S*. *cerevisiae* strains: (15) Delete and prosper, (16) Chemical mutagenesis, (17) Breeding with natural variation, (18) Simply Metabolism, (19) Adaptive evolution with mating, (20) *S*. *cerevisiae* Manual chemostat, (21) Foodie-evolution, (22) *S*. *cerevisiae* Daily dilution, (23) Combined chemostat and temperature fluctuations, (24) Engineering of cold response genes using CRISPR/Cas9, (25) cycles of random mutagenesis with selection, (26) Mating, (27) Ty-induced evolution, (28) Antarticold, (29) Catching cold RNA, (30) *S*. *cerevisiae* temperature gradient. Data for this figure can be found in S2 Data.

Different strains featured various levels of improvement in different growth phase parameters (Fig 3D–3G). Because it is not clear which of the parameters mostly effected ultimate evolutionary success, we conducted a pooled competition experiment to evaluate fitness of each strain at the presence of all others. We competed all bacteria and separately all yeast strains for up to 60 generations, employing a conventional routine of daily dilution into a fresh medium. Competition was done in rich media at the designated low temperature (either 20°C or 15°C for *E*. *coli* and *S*. *cerevisiae*, respectively). We then sequenced the barcode region of the strains in generation 0, 20, 40, and 60 to follow changes in frequency over time. We estimated the fitness of each strain using a published algorithm [19] [20]. The pooled competition results show that one evolved *E*. *coli* variant (strain #7, the “Variable mutation-rate selection” strategy) and one evolved *S*. *cerevisiae* variant (strain #17, the “Breeding with natural variation” strategy) took over the population, both in the organizer’s lab and the other two labs that performed the competition, hence, having the highest fitness based on the pooled competition (Fig 4A and S2 Fig). Having measured separately each strain’s growth parameters allowed us to mathematically simulate and predict the results of the competition in this coculture experiment. This computational procedure employs an extension of the logistic growth equation (see S3 Text). This model assumes competition between strains on shared resources in the coculture, and it assumes otherwise no direct effects between strains, negative (e.g., killing) or positive, which were indeed forbidden in the original call for the Evolthon Challenge. It was encouraging to see that these in silico predictions agree very well with the results of the pooled competition (S3 Fig), indicating that largely, indeed, strains did not affect one another directly in this competition setup.

Because one yeast strain (strain #17, “Breeding with natural variation” strategy) took over the population, we could not rank, in its presence, the rest of the strains. We thus removed this strain from the pool and repeated a competition between all other strains. The winner in this event was strain #26, a “Mating” strategy, another strategy that exercised sexual mating (S4A Fig, lower panel). A third strategy that utilized mating, strategy #19, “Adaptive lab evolution with mating,” obtained the second highest estimated yield in the individual growth curves analysis (Fig 3C and Table 1). Therefore the three fittest yeast strains, coming from three independent labs, were those that utilized sexual reproduction as a means to evolve. In particular, these strategies mated the Evolthon strain with phenotypically diverse natural isolates of yeast strains. They either selected a natural isolate prior to mating, based on growth advantage in cold (strains #19 and #26), or they mated the Evolthon strain with a library of wild isolates, selecting for growth advantage in cold after mating (strain #17). The success of the mating-based strategies in yeast can be rationalized because sex is very well known to improve adaptation because it allows the evolving populations to recombine beneficial mutations that would have otherwise segregated in different populations [21]. In contrast to the success of mating-based strategies, several strategies that used DNA transformations of various sorts (see Table 1) did not fare very well.

In bacteria, the winning strategy, strategy #7, the “Variable mutation-rate selection” strategy, used a high mutation rate using an error-prone DNA polymerase, which was induced at alternative levels. The best colony from the combined evolutionary repeats was chosen for submission. Here, too, we later removed the winner strain and repeated the competition in order to reveal the second highest (S4A Fig, Upper panel). In this case, the winner was a strategy termed “Survival of the fittest group by means of selection” (strain #6) that employed a more complex population genetics approach (the approach utilizes directional selection while increasing the number of tested genotypes; see S1 Table and S2 Text).

In both *E*. *coli* and yeast, we had a strategy termed “daily dilution,” in which cells were daily transferred to fresh media while growing in the same conditions of the final competition. This strategy is commonly used in many lab evolution experiments. These strategies were among the poorest to evolve (Table 1, and S5 Fig). These results indicate that the Evolthon collection of lab evolution strategies likely consists of many useful new means to conduct experiments in evolution in the lab that could improve evolution of microorganisms.

To control for possible biases originating from slight technical differences between labs and in order to examine the robustness of the competition results, we performed the pooled competition experiment under the low temperature conditions in two additional Evolthon labs (one for *E*. *coli* and the other for yeast) using different shakers, incubators, etc. and repeated the barcode sequencing-based fitness measurements, as described above. The results were highly correlated between these repeats, indicating that the results reflect the true ranking of the strains (S2 Fig).

## Revealing trade-offs, memory, and generalization upon adaptation

Evolution often trades off between competing tasks. For example, when improving fitness towards a certain challenge under selection, organisms might compromise their fitness in another environment, in particular the original environment to which they were already adapted. Are there evolutionary strategies that intensify or weaken such trade-offs compared to others? We utilized our set of evolved strains to examine trade-offs by competing the strains in different conditions that were not revealed to the various participating labs when the challenge was announced. The three conditions that we chose were (1) performance at extreme temperature conditions (“extremity”)—here we sought to assess how well each strain performs at an even lower temperature of 8°C; (2) trade-off between evolutionary change and previous legacy (”evolutionary memory”)—Here we were interested in assessing whether evolution toward low temperature compromised the fitness at the original “comfort-zone” temperature (37°C and 30°C for *E*. *coli* and yeast, respectively); (3) performance under a different stressor (“generalization”)—here, we wanted to test whether strains that evolved toward one stress, low temperature, have also gained adaptation, perhaps as an evolutionary by-product, to another stress, an osmotic stress, using NaCl for *E*. *coli* and sorbitol for yeast.

The results in yeast were very clear: the sexually reproducing strain (strain #17, “Breeding with natural variation”) outperformed all others under each of the three additional conditions (Fig 4B lower panel). Here, too, we repeated the competitions in the different conditions without the winning strain (strain #17) to try and reveal other dynamics. The winning strain in the original low temperature challenge condition, i.e., mating strategy #26, won in all other conditions, demonstrating the benefit of sexual reproduction on evolution for unforeseen challenges too (S4B Fig, lower panel). In bacteria, the situation was more complex (Fig 4B upper panel). The winner in the generalization condition was strain #6, “Survival of the fittest group by means of selection,” but in extremity conditions, both strain #6 and strain #7, “Variable mutation-rate selection” (the winner of the completion at 15°C), were the best strains. The behavior of the “Variable mutation-rate selection” strategy across the conditions was interesting. Although this strategy performed the best under the designated low temperature conditions, it did worse in the other, unforeseen challenges. This behavior might indicate that a mutagen can be beneficial in finding a good genetic solution to a particular environment under selection but might compromise other parts of the genome that are presently not under selection but that might prove crucial in the future. As done in yeast, here, too, we removed the winner strain of the challenge condition (strain #7) and repeated the competition in all conditions. The second best strain (strain #6) in the challenge condition, which is the best strain in the generalization condition, won in all conditions once we removed strain #7 (S4B Fig, upper panel).

## Discussion

Evolthon was the first community challenge in lab evolution. It was successful in engaging many labs, mainly through the independent work of students that were very creative, though often employing “backyard biology” in the lab. The joint work of many labs brought two essential assets. First, the strategies chosen were very diverse, highly creative, and they open many new possibilities for new developments. As can be seen in Fig 2, many potential combinations of strategies were not explored (here) so far. Many additional degrees of freedom may still be utilized. Second, in terms of number and heterogeneity of approaches experimented here, such a community effort can much exceed the scale that is typically achievable by individual researchers and students.

It is also important to note a central limitation of Evolthon and community challenges of this sort. Due to the very nature of this mode of science making, it cannot, and probably should not, attempt to cover and examine systematically all possible parameters and degrees of freedom in the space of strategies. For example, if a conventional research was aimed at finding the concentration of a mutagen that maximizes evolutionary adaptation, typically a single researcher in one lab, they would have carried out an orderly experiment with appropriate controls in which a whole range of concentrations were examined. However, natural evolution actually works the “Evolthon way” in the sense that genomes never evolve by systematically varying their parameters over a range of potential values (say, expression level of a gene or affinity of an enzyme to a substrate). Instead, evolution tries out sporadic solutions and continues with the fittest. In that respect, we might say that here we apply the nature of the evolutionary process to the study of evolution itself.

The conceptual directions revealed here could be important for other fields of biology. For example, in biotechnology, optimal evolutionary strategies are important. It is a common practice to use lab evolution to evolve strains with desired applied properties, such as degradation of biological products [22,23], production of products [24], etc. The search for optimal strategies can lead towards efficient means to screen the parameter space of evolutionary strategies.

In clinical applications, such as in infectious diseases and cancer, it is crucial that the cells will not evolve resistance. The regimen of application of drugs could enhance, or perhaps suppress, evolution of resistance. Can efforts of the type conducted here reveal anti-evolution regimes, e.g., for drug application, that would allow on one hand effective treatment and, on the other, would limit the capacity of the attacked cells to evolve resistance? Perhaps the least efficient strategies tried here could be most useful in this opposite challenge.

In Natural ecologies, when species compete in nature, they often feature an evolutionary arms race. For example, when they attack one another with antibiotics, or when immune cells attack pathogens, they may have evolved to limit the capacity of the other side to evolve resistance to the treatment. The type of thinking presented here could be developed further to ask which attack strategies are more or less prone to allow evolution of resistance by the other side. It would be interesting if attack strategies that appear in nature tend to be those to which resistance is harder to evolve.

**Box. Building the Evolthon community and future challenges**

Many participants of Evolthon met in the Genome Evolution conference, presented their strategies, and discussed the future of the project towards establishing a community in lab evolution. Managing such a community is both enjoyable and challenging. There were several values that are already shared by members of this community: (i) Transparency and sharing: All protocols, results, raw data, and processed files were shared early on between all participants so that everyone can process and analyze the data and reach conclusions independently. The strains themselves are sharable, too, now with the broader scientific community. (ii) Effective communication: Many of the Evolthon participants have met in a broader conference to discussed their strategies and future challenges. A lot of the community organization was done through email, and a special challenge was the analysis of data by all authors and writing this paper. A considerable amount of effort went into the coordination of these activities, and the organizer’s lab had to take a coordinating role. A Facebook page was setup where members can share advice, comments, ask questions, etc. (iii) Independence: All participants were totally independent in thinking about their strategies; no advice or tasks were given ahead of time. A particular issue was the possibility that due to differences in the experimental setup in each lab (e.g., shakers, incubators, etc.), performance in the pooled competition at the organizer’s lab would reflect the similarity between these conditions in different labs. By repeating the competitions in two additional labs we could alleviate this concern. The organizer’s lab took upon itself to perform a “Lenski-style” strategy to ensure that the most commonly used mode of lab evolution is being explored here. All participants took care of their own budget, but the organizer’s lab covered the pooled competition sequencing costs. (iv) Exact detailed guidance: The call for Evolthon (S1 Text) was very detailed, specifying not only the challenge but the exact conditions in which experiments will be done.

Our experience also raised several points one should consider when organizing this type of community efforts. One is the issue of which platforms to use in order to publish the event such that it will reach as many members of the community as possible. Another point to consider is the amount of instruction and limitation to be imposed on the participants. An ideal scenario should allow creativity on hand, while controlling for technical variation between labs that would be necessary for valid comparisons of performance. The characteristics of the chosen challenge should also be considered; on one hand the challenge should be broad enough to allow many types of solutions; on the other hand, it should be simple enough to be accessible to many labs and students.

Finally, we call upon the community of evolutionary biologists to keep developing this arena of mutual exploration in experimental evolution. For example, in a brainstorming session at the 2016 Genome Evolution Conference, the community discussed the next Evolthon challenges. One challenge that emerged was to evolve cells (bacteria, yeast, mammal) towards more efficient heterologous expression of a foreign gene, e.g., human antibodies, hormones, etc. We invite current and prospective members of this community to express their opinions on future challenges towards launching one in near future, on the forum in Evolthon web page (https://evolthon2016.wixsite.com/home/forum-1/the-next-evolthon).

## Supporting information

S1 Fig Growth parameters are correlated in yeast but not in bacteria. Growth parameters (lag, growth rate, and yield) were calculated based on a mathematical model for growth (for details, see S3 Text). Correlations between each two parameters are shown separately for *Escherichia coli* (A–C) and *Saccharomyces cerevisiae* (D–F). Correlation coefficient and statistical significance were calculated based on Pearson correlation and are presented for each plot. Data for this figure was taken from Table 1.
(TIF)

S2 Fig Pooled competition in different labs. (A–B) Strains were mixed and grown for several dozens of generations in serial dilution regimes under (A) 15°C for *Escherichia coli* and (B) 20°C for *Saccharomyces cerevisiae* (see S3 Text for details) in either the organizer’s lab (bottom panel) or a different lab (upper panel). At different time points during the competition, barcodes were sequenced and their frequencies are shown. (C–D) Strains’ fitness were calculated based on maximum likelihood algorithm (see S3 Text) based on both pooled competition in organizer’s lab or in the other lab. Pearson correlation between the fitness of all strains in both competition assays are shown for either *E*. *coli* (C) or *S*. *cerevisiae* (D). Strains for which <10 reads were measured were not used for fitness and correlation calculations. Strains key: *E*. *coli strains*: (1) Growth advantage in stationary phase; (2) *E*. *coli* Manual chemostat; (3) Saltation-selection and vice versa; (4) Pop-Gen, (5) *E*. *coli* Daily dilution; (6) Survival of the fittest group by means of selection; (7) Variable mutation-rate selection; (8) Variable mutation-rate selection (with cold-shock); (9) Saltation-selection and vice versa; (10) Lazy man; (11) Accelerated Evolution; (12) Strength through diversity: the United States of *E*.*coli* (U.S.E); (13) Combined chemostat and temperature fluctuations, (14) Hypermutation evolution. *S*. *cerevisiae strains*: (15) Delete and prosper; (16) Chemical mutagenesis; (17) Breeding with natural variation; (18) Simply Metabolism; (19) Adaptive evolution with mating; (20) *S*. *cerevisiae* Manual chemostat; (21) Foodie-evolution; (22) *S*. *cerevisiae* Daily dilution; (23) Combined chemostat and temperature fluctuations; (24) Engineering of cold response genes using CRISPR/Cas9; (25) cycles of random mutagenesis with selection; (26) Mating; (27) Ty-induced evolution; (28) Antarticold; (29) Catching cold RNA; (30) *S*. *cerevisiae* temperature gradient. Data for this figure can be found in S2 Data.
(TIF)

S3 Fig In silico competition based on parameters extracted from growth curves. In order to predict the pooled competition results based on the individual growth curves, we have used a simulation that is based on an expanded form of the logistic equation (see S3 Text). The simulation uses the parameters extracted from the individual growth curves done in 20°C and 15°C (*Escherichia coli* and *Saccharomyces cerevisiae*, respectively). (A) A prediction done to *E*. *coli*; (B) a prediction done to *S*. *cerevisiae*. Color bar represents frequency of each strain out of total number of bacteria or yeast Strains key: *E*. *coli strains*: (1) Growth advantage in stationary phase; (2) *E*. *coli* Manual chemostat; (3) Saltation-selection and vice versa; (4) Pop-Gen, (5) *E*. *coli* Daily dilution; (6) Survival of the fittest group by means of selection; (7) Variable mutation-rate selection; (8) Variable mutation-rate selection (with cold-shock); (9) Saltation-selection and vice versa; (10) Lazy man; (11) Accelerated Evolution; (12) Strength through diversity: the United States of *E*.*coli* (U.S.E); (13) Combined chemostat and temperature fluctuations, (14) Hypermutation evolution. *S*. *cerevisiae strains*: (15) Delete and prosper; (16) Chemical mutagenesis; (17) Breeding with natural variation; (18) Simply Metabolism; (19) Adaptive evolution with mating; (20) *S*. *cerevisiae* Manual chemostat; (21) Foodie-evolution; (22) *S*. *cerevisiae* Daily dilution; (23) Combined chemostat and temperature fluctuations; (24) Engineering of cold response genes using CRISPR/Cas9; (25) cycles of random mutagenesis with selection; (26) Mating; (27) Ty-induced evolution; (28) Antarticold; (29) Catching cold RNA; (30) *S*. *cerevisiae* temperature gradient. Data for this figure can be found in S3 Data
(TIF)

S4 Fig Pooled competition of all strains excluding the most fittest strains. All strains (except for strain “Mating” in *Saccharomyces cerevisiae*, and strain “Variable mutation-rate selection” in *Escherichia* coli) were mixed and grown for several dozens of generations in serial dilution regime under different growth conditions (see S3 Text for details). At different time points during competition, barcodes were sequenced, and their frequencies are shown. (A) Challenge conditions to which strains were evolved (20°C and 15°C for *E*. *coli* and *S*. *cerevisiae*, respectively). Color bar represents the frequency of the strain’s barcode reads from total number of reads. (B) Other challenges include “evolutionary memory,” 37°C and 30°C for *E*. *coli* and *S*. *cerevisiae*, respectively; “generalization,*”* 0.8M NaCl and 1.2M sorbitol for *E*. *coli* and *S*. *cerevisiae*; “extremity” 8°C for both *E*. *coli* and *S*. *cerevisiae*. Color bar represents the frequency of the strain’s barcode reads from total number of reads. Upper panel presents competition results for *E*. *coli*; Lower panel presents competition results for *S*. *cerevisiae*. Strains key: *E*. *coli strains*: (1) Growth advantage in stationary phase; (2) *E*. *coli* Manual chemostat; (3) Saltation-selection and vice versa; (4) Pop-Gen, (5) *E*. *coli* Daily dilution; (6) Survival of the fittest group by means of selection; (7) Variable mutation-rate selection; (8) Variable mutation-rate selection (with cold-shock); (9) Saltation-selection and vice versa; (10) Lazy man; (11) Accelerated Evolution; (12) Strength through diversity: the United States of *E*.*coli* (U.S.E); (13) Combined chemostat and temperature fluctuations, (14) Hypermutation evolution. *S*. *cerevisiae strains*: (15) Delete and prosper; (16) Chemical mutagenesis; (17) Breeding with natural variation; (18) Simply Metabolism; (19) Adaptive evolution with mating; (20) *S*. *cerevisiae* Manual chemostat; (21) Foodie-evolution; (22) *S*. *cerevisiae* Daily dilution; (23) Combined chemostat and temperature fluctuations; (24) Engineering of cold response genes using CRISPR/Cas9; (25) cycles of random mutagenesis with selection; (26) Mating; (27) Ty-induced evolution; (28) Antarticold; (29) Catching cold RNA; (30) *S*. *cerevisiae* temperature gradient. Data for this figure can be found in S2 Data.
(TIF)

S5 Fig In silico pairwise competition simulation of daily dilution strategy against every other strategy. In order to assess the fitness of each strategy relative to that of the daily dilution strategy, we have used a simulation that is based on an expanded form of the logistic equation (see S3 Text). The simulation uses the parameters extracted from the individual growth curves done in 20°C and 15°C (*Escherichia coli* and *Saccharomyces cerevisiae*, respectively). Each subplot shows the results for a single strategy (lower bar) compared to that of daily dilution (upper bar): (A) *E*. *coli*, (B) *S*. *cerevisiae*. Color bar represents the number of cells from each strategy during in silico pairwise competition. Data for this figure can be found in S3 Data.
(TIF)

S1 Text Evolthon announcement file. (PDF)

S2 Text Participants’ materials and methods. Each strategy in Evolthon can be found in this file, including the rational of the strategy, a short description, and a full material and methods.
(DOCX)

S3 Text Materials and methods. This file contains all materials and methods for the paper that were done in the organizer’s lab.
(DOCX)

S1 Table All participants and strategies. (DOCX)

S1 Data Growth experiments. All data, including all repetitions, are found in this file. This file also includes all processed data of the growth experiments, including means and SDs for each time point of the growth curves shown in Fig 3. SD, standard deviation.
(XLSX)

S2 Data Pooled competitions. Read counts for all pooled competition experiment (shown in Fig 4, S2 Fig, and S4 Fig), including all repetitions are found in this Excel file. Each figure data are found in a different sheet.
(XLSX)

S3 Data Simulations. Data for the two simulations (shown in S3 Fig and S5 Fig) are found in this file in different sheets.
(XLSX)

## Abbreviations

DREAM: Dialogue for Reverse Engineering Assessments and Methods
iGEM: International Genetically Engineered Machine
OD: optical density
