# Supplementary figures and images for: Evolthon: A community endeavor to evolve lab evolution
Source: PLoS Biol. 2019 Mar 29;17(3):e3000182. doi: 10.1371/journal.pbio.3000182 (PMC6440615; doi:10.1371/journal.pbio.3000182)

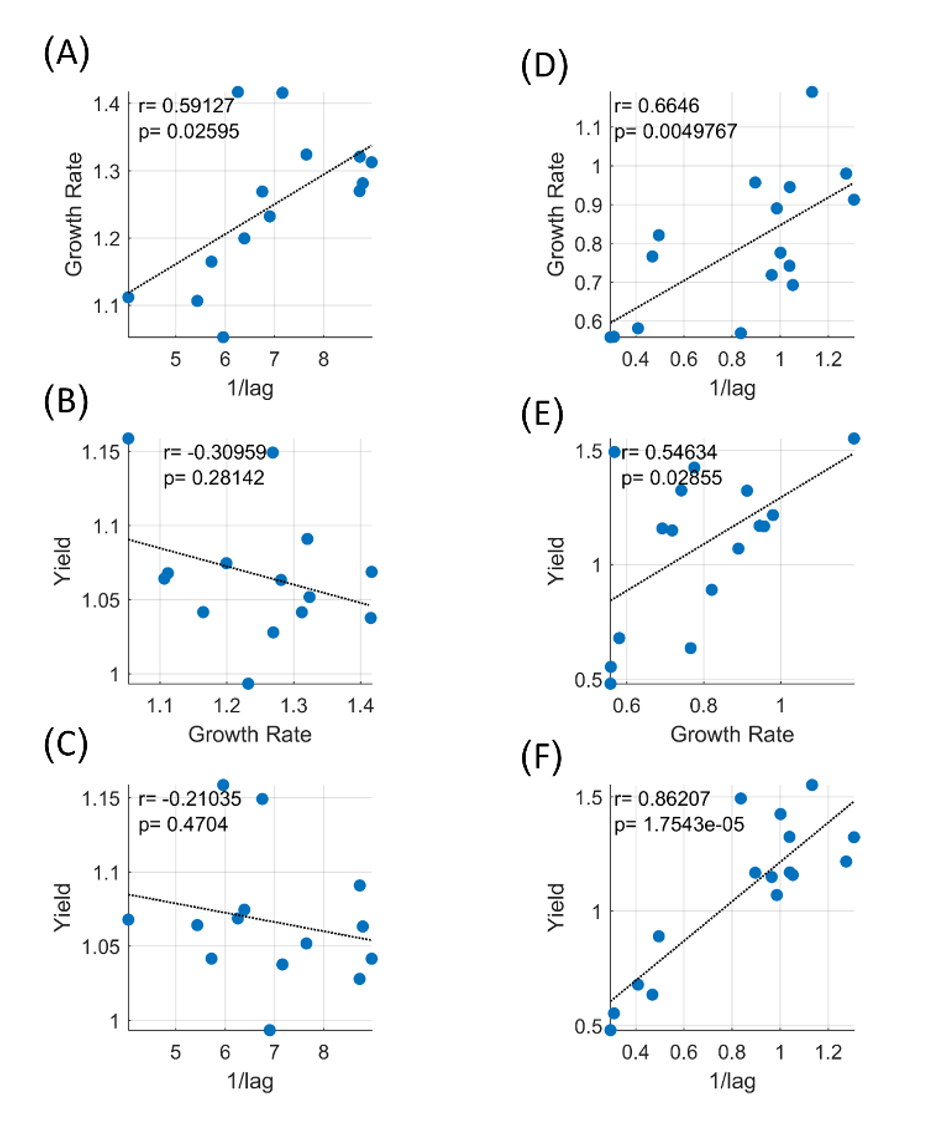

Supplement: S1 Fig — Growth parameters (lag, growth rate, and yield) were calculated based on a mathematical model for growth (for details, see S3 Text). Correlations between each two parameters are shown separately for Escherichia coli (A–C) and Saccharomyces cerevisiae (D–F). Correlation coefficient and statistical significance were calculated based on Pearson correlation and are presented for each plot. Data for this figure was taken from Table 1. (TIF) [file pbio.3000182.s001.tif]

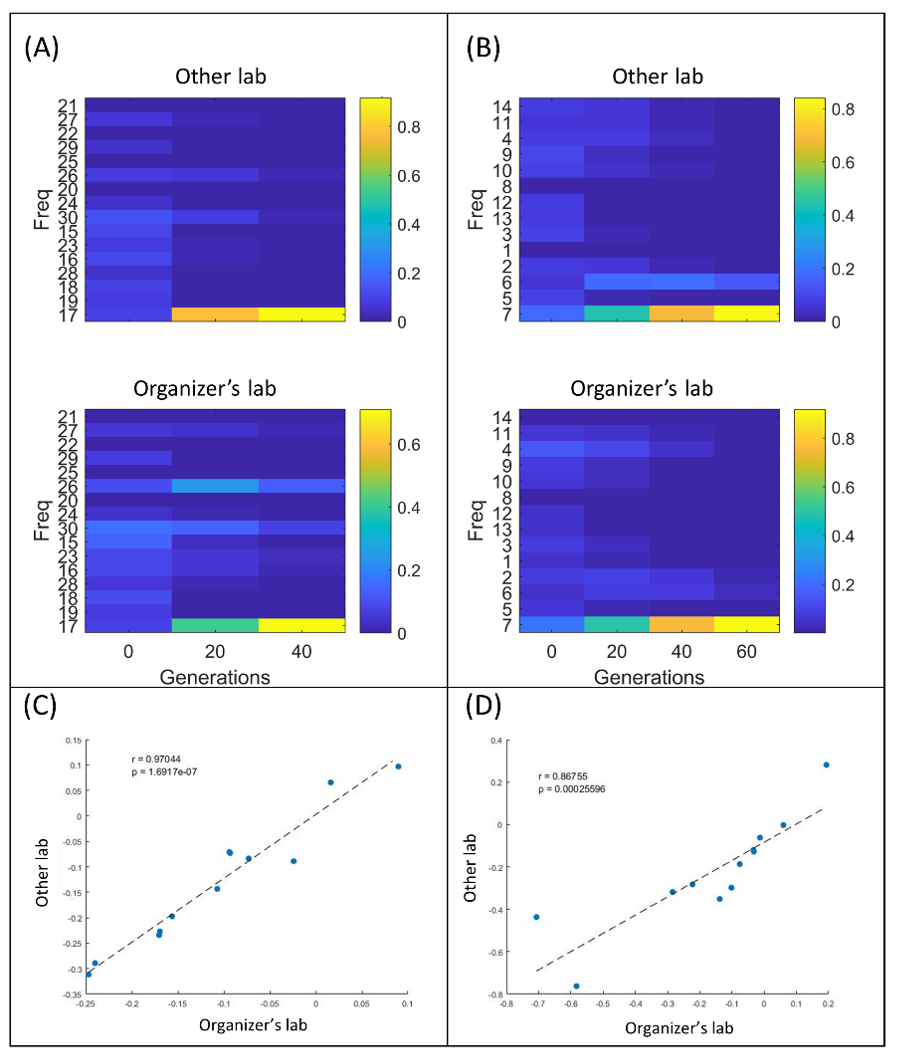

Supplement: S2 Fig — (A–B) Strains were mixed and grown for several dozens of generations in serial dilution regimes under (A) 15°C for Escherichia coli and (B) 20°C for Saccharomyces cerevisiae (see S3 Text for details) in either the organizer’s lab (bottom panel) or a different lab (upper panel). At different time points during the competition, barcodes were sequenced and their frequencies are shown. (C–D) Strains’ fitness were calculated based on maximum likelihood algorithm (see S3 Text) based on both pooled competition in organizer’s lab or in the other lab. Pearson correlation between the fitness of all strains in both competition assays are shown for either E. coli (C) or S. cerevisiae (D). Strains for which <10 reads were measured were not used for fitness and correlation calculations. Strains key: E. coli strains: (1) Growth advantage in stationary phase; (2) E. coli Manual chemostat; (3) Saltation-selection and vice versa; (4) Pop-Gen, (5) E. coli Daily dilution; (6) Survival of the fittest group by means of selection; (7) Variable mutation-rate selection; (8) Variable mutation-rate selection (with cold-shock); (9) Saltation-selection and vice versa; (10) Lazy man; (11) Accelerated Evolution; (12) Strength through diversity: the United States of E.coli (U.S.E); (13) Combined chemostat and temperature fluctuations, (14) Hypermutation evolution. S. cerevisiae strains: (15) Delete and prosper; (16) Chemical mutagenesis; (17) Breeding with natural variation; (18) Simply Metabolism; (19) Adaptive evolution with mating; (20) S. cerevisiae Manual chemostat; (21) Foodie-evolution; (22) S. cerevisiae Daily dilution; (23) Combined chemostat and temperature fluctuations; (24) Engineering of cold response genes using CRISPR/Cas9; (25) cycles of random mutagenesis with selection; (26) Mating; (27) Ty-induced evolution; (28) Antarticold; (29) Catching cold RNA; (30) S. cerevisiae temperature gradient. Data for this figure can be found in S2 Data. (TIF) [file pbio.3000182.s002.tif]

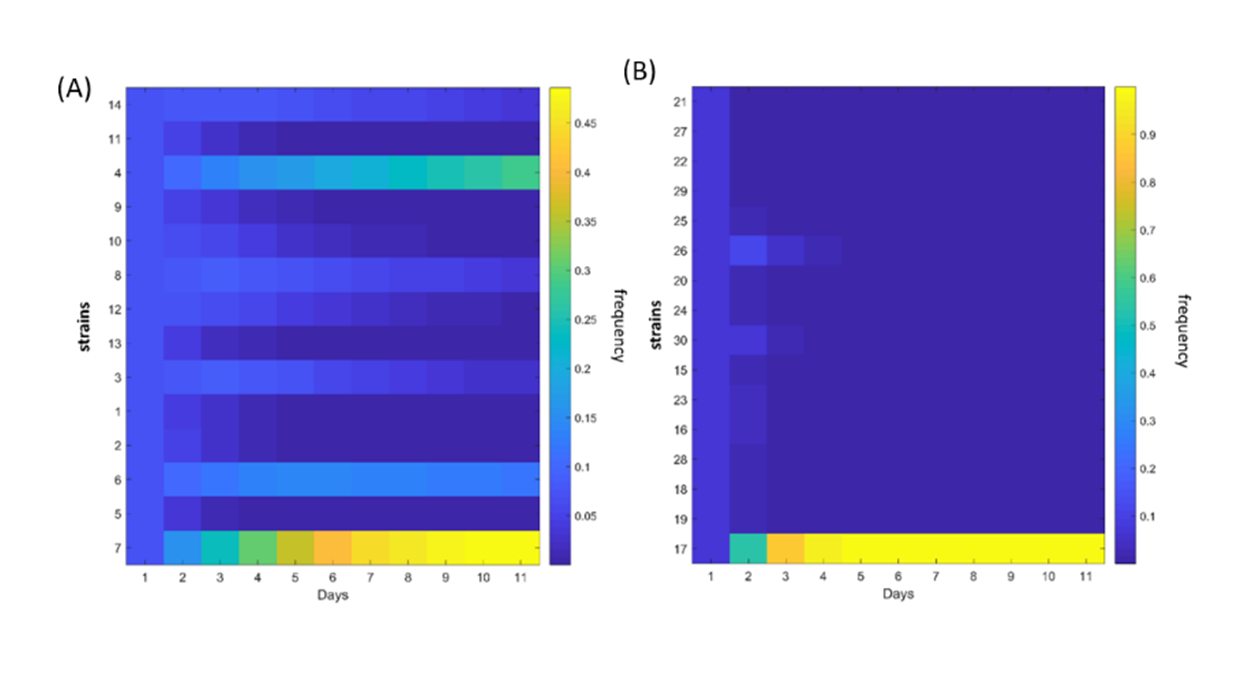

Supplement: S3 Fig — In order to predict the pooled competition results based on the individual growth curves, we have used a simulation that is based on an expanded form of the logistic equation (see S3 Text). The simulation uses the parameters extracted from the individual growth curves done in 20°C and 15°C (Escherichia coli and Saccharomyces cerevisiae, respectively). (A) A prediction done to E. coli; (B) a prediction done to S. cerevisiae. Color bar represents frequency of each strain out of total number of bacteria or yeast Strains key: E. coli strains: (1) Growth advantage in stationary phase; (2) E. coli Manual chemostat; (3) Saltation-selection and vice versa; (4) Pop-Gen, (5) E. coli Daily dilution; (6) Survival of the fittest group by means of selection; (7) Variable mutation-rate selection; (8) Variable mutation-rate selection (with cold-shock); (9) Saltation-selection and vice versa; (10) Lazy man; (11) Accelerated Evolution; (12) Strength through diversity: the United States of E.coli (U.S.E); (13) Combined chemostat and temperature fluctuations, (14) Hypermutation evolution. S. cerevisiae strains: (15) Delete and prosper; (16) Chemical mutagenesis; (17) Breeding with natural variation; (18) Simply Metabolism; (19) Adaptive evolution with mating; (20) S. cerevisiae Manual chemostat; (21) Foodie-evolution; (22) S. cerevisiae Daily dilution; (23) Combined chemostat and temperature fluctuations; (24) Engineering of cold response genes using CRISPR/Cas9; (25) cycles of random mutagenesis with selection; (26) Mating; (27) Ty-induced evolution; (28) Antarticold; (29) Catching cold RNA; (30) S. cerevisiae temperature gradient. Data for this figure can be found in S3 Data (TIF) [file pbio.3000182.s003.tif]

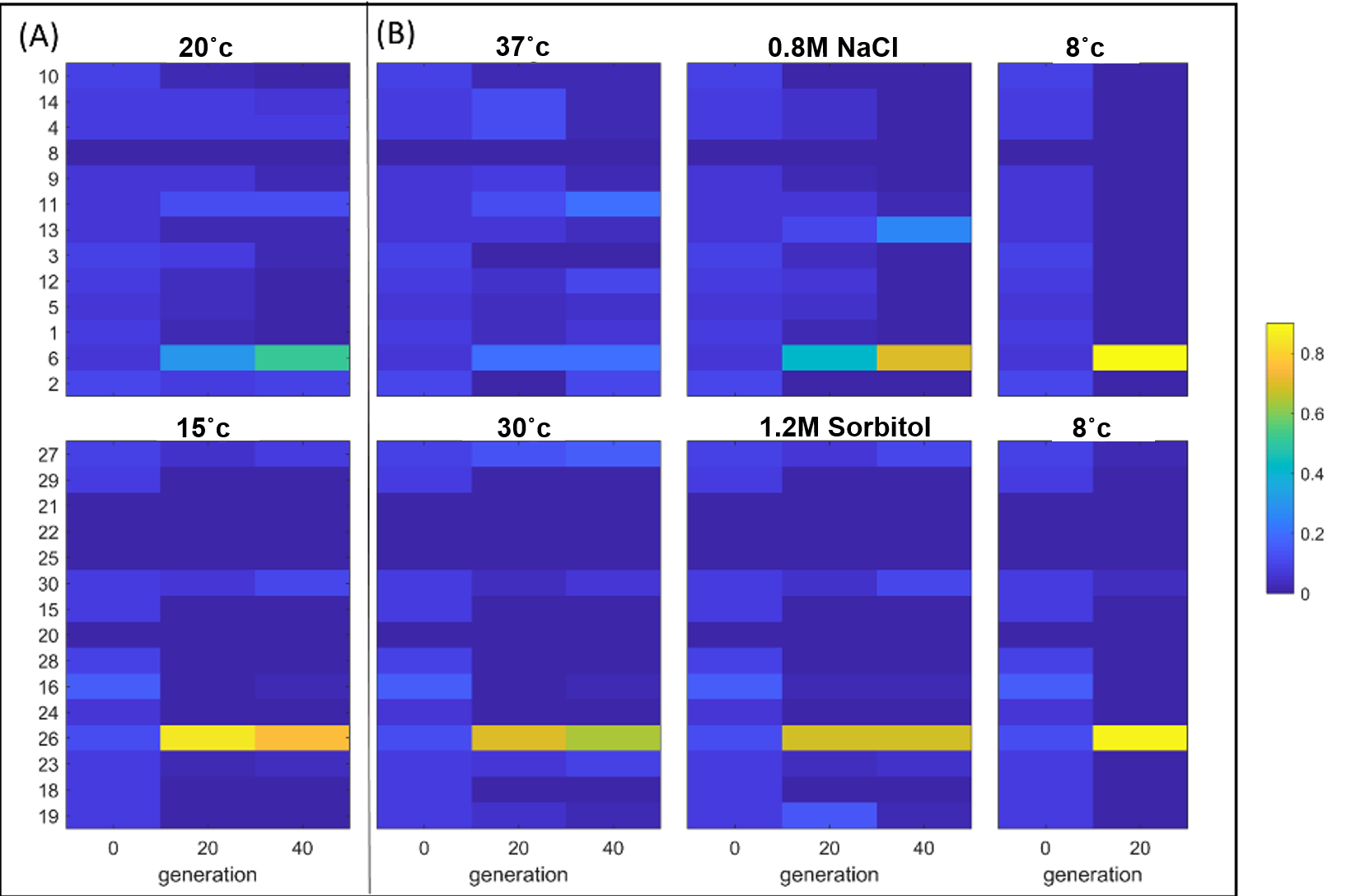

Supplement: S4 Fig — All strains (except for strain “Mating” in Saccharomyces cerevisiae, and strain “Variable mutation-rate selection” in Escherichia coli) were mixed and grown for several dozens of generations in serial dilution regime under different growth conditions (see S3 Text for details). At different time points during competition, barcodes were sequenced, and their frequencies are shown. (A) Challenge conditions to which strains were evolved (20°C and 15°C for E. coli and S. cerevisiae, respectively). Color bar represents the frequency of the strain’s barcode reads from total number of reads. (B) Other challenges include “evolutionary memory,” 37°C and 30°C for E. coli and S. cerevisiae, respectively; “generalization,” 0.8M NaCl and 1.2M sorbitol for E. coli and S. cerevisiae; “extremity” 8°C for both E. coli and S. cerevisiae. Color bar represents the frequency of the strain’s barcode reads from total number of reads. Upper panel presents competition results for E. coli; Lower panel presents competition results for S. cerevisiae. Strains key: E. coli strains: (1) Growth advantage in stationary phase; (2) E. coli Manual chemostat; (3) Saltation-selection and vice versa; (4) Pop-Gen, (5) E. coli Daily dilution; (6) Survival of the fittest group by means of selection; (7) Variable mutation-rate selection; (8) Variable mutation-rate selection (with cold-shock); (9) Saltation-selection and vice versa; (10) Lazy man; (11) Accelerated Evolution; (12) Strength through diversity: the United States of E.coli (U.S.E); (13) Combined chemostat and temperature fluctuations, (14) Hypermutation evolution. S. cerevisiae strains: (15) Delete and prosper; (16) Chemical mutagenesis; (17) Breeding with natural variation; (18) Simply Metabolism; (19) Adaptive evolution with mating; (20) S. cerevisiae Manual chemostat; (21) Foodie-evolution; (22) S. cerevisiae Daily dilution; (23) Combined chemostat and temperature fluctuations; (24) Engineering of cold response genes using CRISPR/Cas9; (25) cycle [file pbio.3000182.s004.tif]

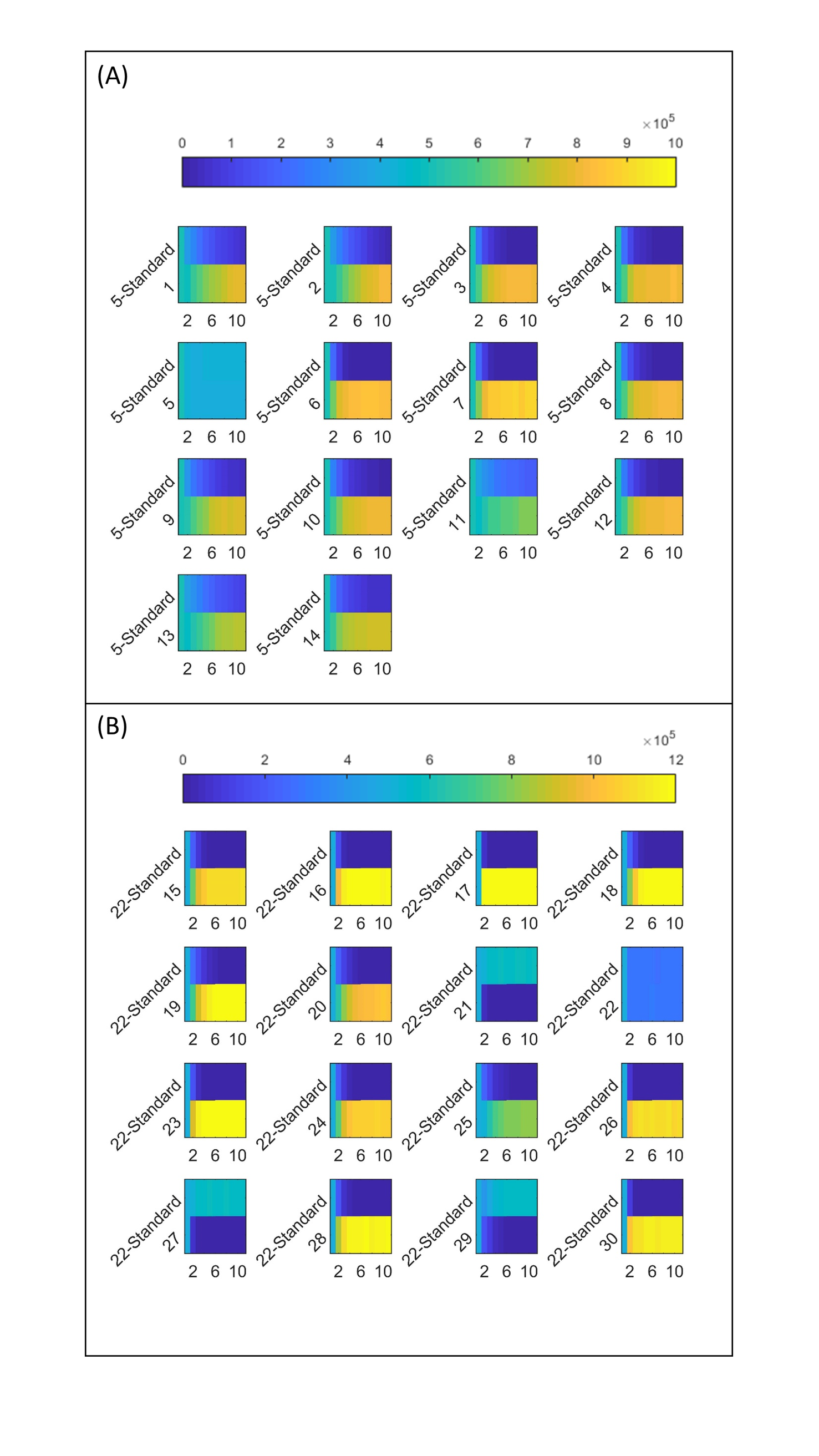

Supplement: S5 Fig — In order to assess the fitness of each strategy relative to that of the daily dilution strategy, we have used a simulation that is based on an expanded form of the logistic equation (see S3 Text). The simulation uses the parameters extracted from the individual growth curves done in 20°C and 15°C (Escherichia coli and Saccharomyces cerevisiae, respectively). Each subplot shows the results for a single strategy (lower bar) compared to that of daily dilution (upper bar): (A) E. coli, (B) S. cerevisiae. Color bar represents the number of cells from each strategy during in silico pairwise competition. Data for this figure can be found in S3 Data. (TIF) [file pbio.3000182.s005.tif]
