# Supplementary material for: Evolthon: A community endeavor to evolve lab evolution
Source: PLoS Biol. 2019 Mar 29;17(3):e3000182. doi: 10.1371/journal.pbio.3000182 (PMC6440615; doi:10.1371/journal.pbio.3000182)
Supplement: S1 Text — (PDF) [file pbio.3000182.s006.pdf]

Evolthon: the lab evolution challenge

We are happy to announce first edition of the **Evolthon**. **Evolthon** is a lab evolution challenge for the community of scientists interested in microbial evolution. We are aiming to find new and creative strategies of microorganisms' adaptation toward a given challenge in a laboratory setting. Evolthon will take place as a part of the "Genome Evolution" meeting at the Weizmann institute of Science on 1st-3rd November 2016.

Participants will be asked to evolve or engineer microorganisms (*S. cerevisiae* or *E.coli*) toward a specific environmental condition – growth at low temperature. We are interested in exploring with the community how various evolutionary regimes and strategies, such as the extent of mutagenesis, population size, existence of sex, severity of stress, status of epigenetic machineries, etc. could affect the trajectory of evolution. We also allow designed engineering of the genome.

We expect a shared publication of an article authored by all interested participants in which we will describe the challenge, the strategies and the results.

Experimental setup: strains, Challenges and results evaluation

The challenge would be growth at low temperature (15°C for yeast and 20°C for *E. coli*). To assess the evolved strains' fitness on the challenging condition, all competing yeast strains will be pooled together, and separately, all competing *E. coli* strains will be pooled together and allowed to compete within each pool in order to assess their relative fitness. The starting strains will be *S. cerevisiae* (strain BY4741), and *E. coli* (strain MG1655). Growth medium in the competition will be YPD for yeast (*10g/l yeast extract, 20g/l peptone, 20g/l glucose*), and LB for *E. coli* (*10g/l tryptone, 5g/l yeast extract, and 10g/l NaCl*).

Rules

1) Strains: Each participant will receive from the organizers a unique genomic barcoded ancestor from the respective species.
2) Mode of competition: All strains from each species will be pooled together and will be grown in batch mode with daily dilution regime at the above temperature and medium for a couple of weeks to allow competition. At the end of this competition period the organizers will deep-sequence the barcodes from the pooled populations to determine the relative fitness of each strain.
3) Submission of strains: each participant will be required to provide the following:
    a. The evolved/engineered strains
    b. A short description of the chosen evolutionary strategy
    c. A publication-ready Materials & Methods documentation of the evolution/engineering process/protocol
    d. Growth curves of ancestor and adapted strains on the challenge's medium and temperature
    e. Whole genome sequencing of the strain (optional, and can be submitted at a later stage).

Registration – 15/4/2016

Submission of strains and information – Sept 1st 2016

Results announcement

Results announcement and discussion will take place at a special session during the Genome Evolution Meeting. Individual participants will be invited to present their strategies.

A joint publication of the challenge

The results will be summarized and submitted as a joint paper authored by all participants who would be interested. Strategies will have to be documented fully and described in the publication. Individual results will not disclose author identity.

Contact

If you are interested to participate please contact Tzachi Pilpel (Pilpel@weizmann.ac.il) AND Ruth Towers (ruth.towers@weizmann.ac.il). Please indicate in which track you are interested – *E. coli* or *S. cerevisiae* and send mailing address for strains shipment.

Adapted strains should be sent to the following address:

Dr. Ruth Towers
Pilpel Lab (Belfer building)
Department of Molecular Genetics
The Weizmann Institute of Science
234 Herzl Street,
Rehovot 7610001
Israel

For further questions please contact Tzachi Pilpel (Pilpel@weizmann.ac.il).
