# Supplementary material for: Evolthon: A community endeavor to evolve lab evolution
Source: PLoS Biol. 2019 Mar 29;17(3):e3000182. doi: 10.1371/journal.pbio.3000182 (PMC6440615; doi:10.1371/journal.pbio.3000182)
Supplement: S2 Text — Each strategy in Evolthon can be found in this file, including the rational of the strategy, a short description, and a full material and methods. (DOCX) [file pbio.3000182.s007.docx]

Contents

[Strategy #1: Growth advantageous in stationary Phase (GASP) 2](#_Toc533082022)

[Strategy #2: *E. coli* Manual chemostat 3](#_Toc533082023)

[Strategy #3 and #9: Saltation-Selection or vice versa 4](#_Toc533082024)

[Strategy #4: Pop-Gen 5](#_Toc533082025)

[Strategy #5: *E. coli* Daily dilution 6](#_Toc533082026)

[Strategy #6: Survival of the fittest by means of directional selection 7](#_Toc533082027)

[Strategies #7 and #8: Variable mutation-rate selection with and without cold-shock 8](#_Toc533082028)

[Strategy #10: Lazy man 9](#_Toc533082029)

[Strategy #11: Accelerated Evolution 10](#_Toc533082030)

[Strategy #12: Strength through diversity: the United States of *E.coli* (U.S.E) 11](#_Toc533082031)

[Strategy #13: Combined chemostat & temperature fluctuations 12](#_Toc533082032)

[Strategy #14: Hypermutation evolution 13](#_Toc533082033)

[Strategy #15: Delete and prosper 14](#_Toc533082034)

[Strategy #16: Chemical mutagenesis 15](#_Toc533082035)

[Strategy #17: Breeding with natural variation 16](#_Toc533082036)

[Strategy #18: Simply Metabolism 17](#_Toc533082037)

[Strategy #19: Adaptive lab evolution with mating 18](#_Toc533082038)

[Strategy #20: *S. cerevisiae* Manual chemostat 19](#_Toc533082039)

[Strategy #21: Foodie-evolution 20](#_Toc533082040)

[Strategy #22: *S. cerevisiae* Daily dilution 21](#_Toc533082041)

[Strategy #23: Combined chemostat & temperature fluctuations 22](#_Toc533082042)

[Strategy #24: Engineering of cold response genes using CRISPR/Cas9 23](#_Toc533082043)

[Strategy #25: Cycles of random mutagenesis with selection 24](#_Toc533082044)

[Strategy #26: Mating 25](#_Toc533082045)

[Strategy #27: Ty-induced evolution 26](#_Toc533082046)

[Strategy #28: Antarticold 27](#_Toc533082047)

[Strategy #29: Catching cold RNA 28](#_Toc533082048)

[Strategy #30: *S. cerevisiae* temperature gradient 29](#_Toc533082049)

#
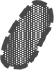

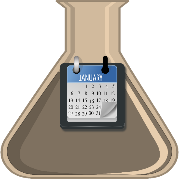
Strategy #1: Growth advantage in stationary Phase (GASP)

Omri Adini, Michael Klutstein, Ronen Hazan

Faculty of Dental Medicine, The Hebrew University of Jerusalem, Jerusalem 9112001, Israel

Description: *E. coli* cells were adapted to growth in a constant temperature stress of 20˚C by long time incubation (2 month) without any transfer or addition of nutrients.

Rationale: Previously it was shown that *E. coli* cells can survive years of starvation without any nutrient addition. Furthermore, the surviving cells exert remarkable tolerance to many stress conditions, including such that they were not expose to {Finkel, 2006 #1;Finkel, 1999 #3;Zambrano, 1996 #2;Zinser, 2004 #4}. This adaptation process was termed Growth advantageous in stationary phase (GASP) {Zambrano, 1996 #2}. The adaptation time was shown to be within few month {Avrani, 2017 #6}. These observations led us to speculate that adaption to cold condition might be achieved via GASP mechanism.

Materials and Methods:

*E. coli* cells were incubated for 2 months in 5ml tubes in LB media under 200rpm shaking at 20˚C without addition of nutrients. At the end of the incubation the bacteria were plated on LB agar plate, incubated in 20˚C until colonies observed and one large colony was picked, regrown and sent to the organizers.

#

#
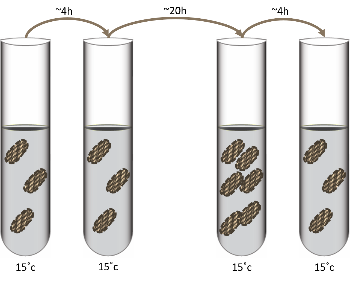

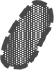
Strategy #2: *E. coli* Manual chemostat

Avihu H. Yona

Physics of Living Systems, Department of Physics, Massachusetts Institute of Technology, Cambridge, MA, 02139, USA

Description: *E. coli* strain was adapted to cold by diluting the cells twice a day.

Rationale: Aiming to reduce lag phase, cells were diluted twice a day, where the second time was couple of hours after the first dilution of the day.

Materials and Methods: Daily dilution of 1:100 was made twice a day into 1ml of fresh LB at 20˚C (on a horizontal shaker). Each day the second dilution was done between 2-8h after the first dilution.

#
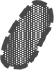
Strategy #3 and #9: Saltation-Selection or vice versa
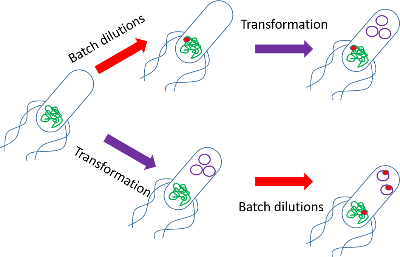


Idan Yelin+, Ning Yin+, Dor Russ+, Einat Tamar+, Olga Snitser+, Daniel Schultz^, Roy Kishony+*

+ Faculty of Biology, Technion – Israel Institute of Technology, Haifa, Israel

^Department of Microbiology and Immunology, Geisel School of Medicine at Dartmouth, Hanover, New Hampshire

*Faculty of Computer Science, Technion–Israel Institute of Technology, Haifa, Israel.

Description and rationale:

**Adding the chaperins GroEL/S and evolving by serial transfer in 20˚C**

A two-pronged strategy: (1) evolutionary *saltation* by acquiring cold-adapted chaperonins and (2) mutation *selection* step in liquid culture. We used these steps in tandem at different orders, both as saltation-selection and selection-saltation, to distinguish how the sequence of evolutionary events affects the final outcome in both magnitude and path.

Materials and Methods:

**Saltation**: GroEL/S (Cpn60/10) chaperonins from the psychrophilic bacterium *Oleispira antarctica* were cloned into a pUC57 plasmid downstream of the *E. coli* endogenous GroEL/S σ32 promoter and ribosome binding site. Bacteria were heat-shock transformed. Transformants were selected with Ampicillin. Ampicillin was not used while growing in cold temperature.

**Selection**: 30 parallel bacterial cultures were grown in a 96-well plate in 150ul LB-Miller (10g/l tryptone, 5g/l yeast extract, 5g/l NaCl) and diluted 1:100 in a 24 hours cycle.

For saltation-selection bacteria were first transformed with the GroEL/S expressing plasmid and then 30 parallel cultures were cultivated for 32 days with daily dilutions. For selection-saltation bacteria were first cultivated for 32 days in 30 parallel cultures with daily dilutions, and only then the fittest cultures, as determined by growth curve measurement, were transformed.

Batch dilution followed by transformation was carried out for strain 3 and vice versa for strain 9.

#
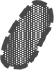

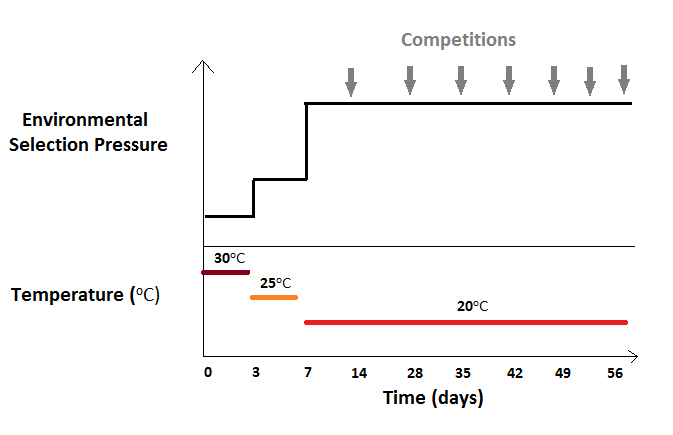
Strategy #4: Pop-Gen

Nilima Walunjkar

Deepa Agashe’s lab, National Centre for Biological Sciences, Bangalore, India

Description: Multiple replicates of the *E. coli* strain were grown in a 48 well plate at 20 degree Celsius and diluted every 24 hours. Competition between the replicates was carried out after every 7 dilutions.

Rationale: This selection regime enriches for mutations that are beneficial for growth and survival at 20 degree Celsius. Competing replicates allows beneficial mutations to fix and new mutations will arise in this background.

Material and Methods:

The *E. coli* strain was initially grown at 30 degree Celsius in a 100 ml conical flask for 3 days and the temperature was lowered to 25 degree Celsius for the next 4 days. Subsequently, 44 populations were started in a 48 well plate containing 600 microliters of LB. These populations were diluted every 24 hours (1:100). This bottlenecking led to some drift in the populations. After 7 dilutions, all the populations in the plate were pooled together to constitute the competition mix. We used this as the inoculum in the next dilution. After 24 hours, these populations were diluted again and the process was repeated. Evolving 44 parallel populations increases the sampling of the mutation space. In the ensuing competition it is expected that the most beneficial mutation outcompetes the others and gets fixed in the population. Our populations went through 56 bottlenecks and had approximately 448 generations. For the final competition, all the populations were combined and evolved in a flask for 3 days at 20 degree Celsius. This culture was streaked out on a LB plate and a single colony was picked for submission.


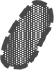


#
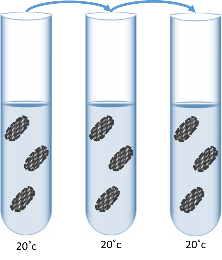
Strategy #5: *E. coli* Daily dilution

Sivan Kaminski Strauss, Orna Dahan, Yitzhak Pilpel

Department of Molecular Genetics, Weizmann Institute of Science, Rehovot, Israel

Description: *E. coli* cells were adapted to growth in a constant temperature stress of 20˚C by daily diluting the evolving *E. coli* population.

Rationale: This strain serves as a base line for evolution under low temperature. By design its evolution was under simplest possible setup that only exposes cells to low temperature.

Materials and Methods:

*E. coli* cells were grown 1.2ml of LB media (5g/L yeast extract, 10g/L tryptone, 10g/L NaCl) in a 24-well plate under constant shaking of ~800rpm at 20˚C. Upon reaching stationary phase (~25 hours) cells were diluted 1:120 into fresh LB media. Evolution was done for ~170 generation in 4 repetitions.

At the end of the evolution, samples from each of the evolving populations were plated on LB-agar plates. Single colonies were picked randomly for growth experiment in a robotic system. Each colony was incubated in liquid LB media and grown for 24-hours at 20˚C while shaking at ~800rpm, OD600 was measured at 30min intervals, and the best performing colony was chosen as the participant strains for Evolthon Challenge.

#
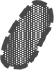


# Strategy #6: Survival of the fittest by means of directional selection


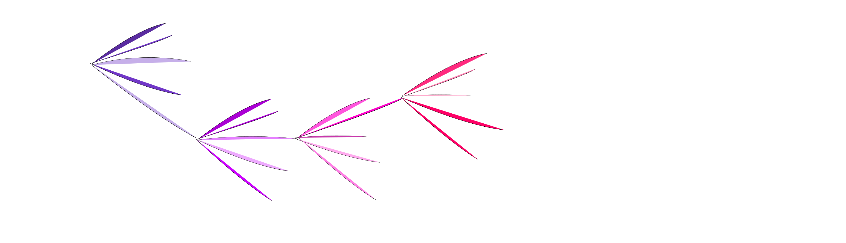


Tanita Wein^1^, Tal Dagan^1^

^1^Institute of Microbiology, Kiel University, 24118 Kiel, Germany

Description

We chose an experimental evolution approach to adapt *E. coli* to cold stress at 20°C. Briefly, five *E. coli* populations were evolved in parallel while repetitively imposing directional selection for the fittest population. The fitness was measured by a growth advantage, which is defined by fastest growth in the exponential growth phase of the populations.

Rationale

An experimental evolution approach has the advantage of applying natural selection for an adaptive trait within the native *E. coli* genetic context (e.g. ref 1,2). Our approach utilizes directional selection while increasing the number of tested genotypes.

Material & Methods

In this study we used *Escherichia coli* K-12 MG1655 containing the nptII gene as a marker inserted at a neutral site in the bacterial chromosome. The ancestral strain was cultured on LB agar plates at 37°C. Three colonies were randomly sampled as the ancestral clones (n=3). The cold adaptation experiment was initiated by inoculating the three clones in 1ml tubes containing LB broth. After overnight incubation at 20°C, the ancestral populations were sampled into five subpopulations of each replicate. The five subpopulations were grown at 20°C with constant orbital shaking of 250 rpm. After 24h, cell density was measured by means of optical density (OD) at 600 nm. The subpopulation having the highest cell number (i.e. the fastest growing) was selected as the ancestor for the next round. We used a dilution of 100-fold into fresh medium allowing an estimated 7 generations of binary fission (log2 of the dilution factor) to maintain a cell density of approximately 109 cells/ml. We repeated the selective propagation 100 times, which amounts to approximately 700 generations. The Evolthon-clone was chosen from the fastest growing subpopulation after 700 generations growth in 20°C.

References

1. Barrick JE, Lenski RE (2013) Genome dynamics during experimental evolution. Nature Reviews Genetics 14:827-839.

2. Burke MK (2012) How does adaptation sweep through the genome? Insights from long-term selection experiments. Proceedings of the Royal Society B: Biological Sciences 279:5029-5038.

#
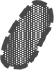

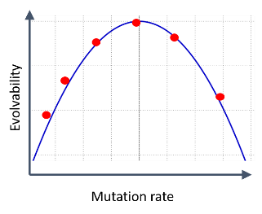
Strategies #7 and #8: Variable mutation-rate selection with and without cold-shock

David G. Wernick, Elad Herz, Yinon Bar-On

Department of Plant and Environmental Sciences, Weizmann Institute of Science, Rehovot 7610001, Israel

**Strain 7**

Description: Cold-evolved strain were generated through continuous selection at approximately 20^0^C with a wide range of artificially-bolstered random mutation rates.

Rationale: there is a trade-off between mutation rate and adaptation. Low mutation rate will cause a static and un-evolved population, while a high mutation rate may cause too many mutation that could be lethal. In this approach we used different rates of mutation to find the best evolved strain.

Material & Methods: The wild-type strain was transformed with mutator plasmid MP6 (a gift from David Liu; Addgene plasmid #69669) containing error-prone DNA polymerase mutant genes of *dnaQ926*, *dam*, *seqA*, *emrR*, *ugi*, and *CDA1* (gene details in reference 1). Differential mutation rates were induced by D-arabinose (Sigma-Aldrich) supplementation at 0, 0.001, 0.01, 0.1, or 1 %w/v, and held constant within individual cultures throughout the evolution. Cells were grown continuously in 2mL LB medium in 14 mL snap-cap culture tubes (BD Biosciences). The LB was supplemented with D-arabinose, kanamycin sulfate (25 ng/ml), and chloramphenicol (15 ng/ml) as appropriate. Tubes were kept in a room with temperature maintained between 19 – 21 ^0^C, and rotating on an open-air platform shaker at 250 rpm. Cultures were routinely diluted 100 to 3,000 fold with fresh medium upon reaching visible turbidity. Chloramphenicol was emitted from the medium for the last four weeks of culturing to support MP6 curing.

At the end of the evolution process one *E. coli* colony was chosen from plate randomly.

**Strain 8**

Description: Cold-evolved strain were generated with a wide range of artificially-bolstered random mutation rates. In addition, a cold shock step was introduced in each dilution.

Rationale: the addition of the cold shock will add high selective pressure, selecting the fittest cells.

Material & Methods: cells were kept in a 4 ^0^C fridge for 60 to 90 min following each dilution with fresh medium and before moving to the 20 ^0^C room. This was maintained throughout the first month of culturing. Additionally, D-arabinose was only supplemented at 0 and 0.01%.

References

1. Development of potent in vivo mutagenesis plasmids with broad mutational spectra. Badran AH, Liu DR. Nat Commun. 2015 Oct 7;6:8425. doi: 10.1038/ncomms9425

#
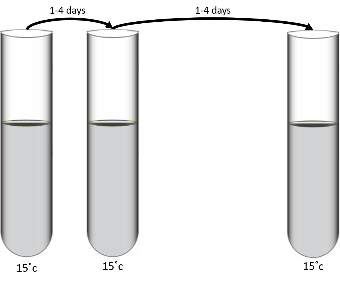

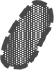
Strategy #10: Lazy man

Atray Dixit

Broad Institute of MIT and Harvard, Cambridge, MA 02142, USA

Harvard-MIT Division of Health Sciences and Technology, Cambridge, MA 02139, USA

Description: Cells were grown at room temperature and diluted once every 1-4 days, semi chaotically.

Rationale: In the semi chaotically routine, I intended to shorten lag phase. Cells had to exit from different levels of stationary phase, since the different time periods spent in stationary may contribute to different lag phase time.

Material and methods:

*E. coli* cells were grown 200ml of LB media (5g/L yeast extract, 10g/L tryptone, 10g/L NaCl) at room temperature (~25˚C). Once every 1-4 days cells were diluted 1:120 into fresh LB media. Evolution was done for ~800 generations.

At the end of the evolution, culture was plated on LB-agar plates. A random single colony was picked and sent to the competition organizers.

#
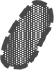

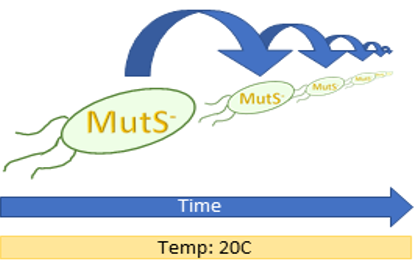
Strategy #11: Accelerated Evolution

Aditya M. Kunjapur*, Timothy M. Wannier*, George M. Church

Department of Genetics, Harvard Medical School, Boston, MA, USA

Description: conferring a hyper-mutator strain

Rationale: high mutation rate will create a strain that adapt faster to stress

Material and Methods:

The plasmid pKD78 with the lambda red machinery (beta/exo/gam) under inducible control of the arabinose promoter was introduced into the strain with chloramphenicol selection [Ref. 1]. This machinery was then induced at mid-log and the following 90-bp oligonucleotide ordered from Integrated DNA Technologies (IDT) to disable mutS was electroporated into the cells:

a*c*cccatgagtgcaatagaaaatttcgacgcccatacgcccatgatgcagcagtgatagtcgctgaaagcccagcatcccgagatcctgc

Transformed cells were recovered overnight, followed by dilution onto agar plates containing chloramphenicol. Colonies were screened by allele-specific PCR to identify successful mutS inactivation using the following oligonucleotides as previously described [Ref. 2]:

| mutS-2_ascPCR_wt-F | CCATGATGCAGCAGTATCTCAG |
| --- | --- |
| mutS-2_ascPCR_mut-F | CCATGATGCAGCAGTGATAGTC |
| mutS-2_ascPCR-R | AGGTTGTCCTGACGCTCCTG |

A colony with disabled mutS was isolated and passaged overnight. This strain was then subjected to lambda red induction several times during one week with the goal of inserting a gene cassette into the endogenous tolC locus on the genome. The cassette contained an artificial operon of cpn10/60 (codon-optimized nucleotide sequence of Oleispira antarctica genes for E. coli synthesized by IDT) [Ref. 3]. Selection against tolC occurred over multiple attempts but failed to produce a strain with the insertion. As a result, the strain was instead directly moved to 20ºC and serially passaged, once per day for approximately six weeks. Passages were conducted as previously reported [Ref. 4] (i.e., at a ratio of 1:1,000 culture volume to fresh media, in a total culture volume of 1 ml in a shaking temperature-controlled incubator) with the exception of using Lennox LB for these passages rather than M9 minimal media.

References:

1. Datsenko, KA, BL Wanner 2000. One-step inactivation of chromosomal genes in Escherichia coli K-12 using PCR products. Proc. Natl. Acad. Sci. U.S.A. 97(12):6640-5.

2. Kunjapur, A. M., Stork, D. A., Kuru, E., Vargas-Rodriguez, O., Landon, M., Söll, D., & Church, G. M. (2018). Engineering posttranslational proofreading to discriminate nonstandard amino acids. Proceedings of the National Academy of Sciences, 115(3), 619-624.

3. Ferrer, M., Chernikova, T. N., Yakimov, M. M., Golyshin, P. N., & Timmis, K. N. (2003). Chaperonins govern growth of Escherichia coli at low temperatures. Nature biotechnology, 21(11), 1266.

4. Wannier, T. M., Kunjapur, A. M., Rice, D. P., McDonald, M. J., Desai, M. M., & Church, G. M. (2018). Adaptive evolution of genomically recoded Escherichia coli. Proceedings of the National Academy of Sciences, 115(12), 3090-3095.

#
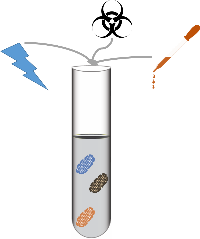

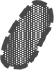
Strategy #12: Strength through diversity: the United States of *E.coli* (U.S.E)

*Shannon Johnson^a,b,c,*^, Simmie Foster^b,e,f^, Timothy Stiles^c^, Noah Jakimo^c^, Thrasyvoulos Karydis^c^,*

*Rachel Soo Hoo Smith^c^, Kate Adamala^d^, Andreas Mershin^c*^*

*^a^*Harvard University Extension School, Cambridge, MA 02138 USA; ^b^Harvard Medical School, Boston, MA 02115 USA; *^c^*Massachusetts Institute of Technology, Center for Bits and Atoms, Cambridge, MA 02913 USA; ^d^University of Minnesota, Minneapolis MN 55108 USA; ^e^Massachussets General Hospital, Boston, MA 02114 USA; ^f^Boston Children’s Hospital, Boston, MA 02115 USA;

Description: We developed a protocol to create diverse populations amounting to diverse origin histories of “multicultural” *E. col*i, each subculture subjected to a different combination of mutagenic stressors, shaping their own unique survival strategy.

Rationale: Our strategy was inspired by a loose, perhaps equal parts art and science analogy to the "population melting pot" of the United States. While at this early stage we can draw no robust anthropomorphic parallels, we were guided by the question of whether a diverse population of *E-coli* cultured under various stressors before being pooled into one culture, would gain the ability to be in general better, i.e. faster and more resilient when faced with adapting to the unknown. Would such a mutli-culture be overall more robust at surviving any challenge when compared to a homogenous population selected to do well in any particular challenge?

Materials and Methods:

Cells were first chemically mutagenized, with each tube receiving a different treatment (or controls with no mutagen), then allowed to grow at 18^o^C to select for adaptive mutations. The presumptive mutants and controls were then plated into 96 well plates and treated with 18 differently graded doses of UV, resulting in 254 different subcultures, each exposed to a unique combination of chemical and UV mutagenesis. These subcultures were again allowed to grow at 18^o^C. The double mutagenized (chemical+UV exposed) bacteria were additionally co-incubated with heat-killed “Fast” bacteria to potentially confer an adaptive advantage. Finally, double mutagenized (chemical+UV), single mutagenized (chemical or UV only), and Original bacteria, representing a total of 613 different subcultures, were pooled to create the “United States of E. Coli” (U.S.E.) culture submitted for the competition.

*Chemical* *Mutagenesis*: Mutagen concentrations, treatment duration, and post-treatment incubation times are listed in the table [1]. Treatment occurred at room temperature (RT). After treatment, mutagenized *E. coli* were recovered before UV treatment.

*Ultraviolet Mutagenesis*: mutagenized cells were diluted 1:1000 in LB and treated in 250ul in 1mL 96-well plates. Each plate had two controls (received no UV exposure), and treatment times were 10seconds, 20s, 40s, 1m, 1.5m, 2m, and every minute up to 14m (18 separate exposure times per chemically mutagenized subculture). UV Exposure was through the wells from the bottom at RT. After exposure, plates were incubated at 37°C for 24hrs then moved to 18°C for 96hrs.

*Fast Bacteria Treatment*: Fast were killed via pasteurization at 75°C for 4.5hrs before the addition of kanamycin to the dead/non-dividing culture. Dead Fast bacteria were added to each subculture. Killed fast bacteria were added 1:5 to mutagenized cells. recovery was done in two steps; they were incubated at 37°C for 23hrs then moved to 20°C for 23hrs.

*Creation of United States of E. Coli Strain and Growth Curve*: 50uL from each of the 612 subcultures, each exposed to a unique combination of chemical, UV, and heat-killed bacteria, was pooled to create the final competition strain (“Mix”). This strain was compared to the Original and Fast. See figure describing growth experiment in [Evolthon site](https://evolthon2016.wixsite.com/home)

**[1]** “A Survey of Chemicals for Mutagenic Action on E. coli,” by M. Demerec, G. Bertani and J. Flint, *The American Naturalist* Vol. 85, No. 821 (Mar. - Apr., 1951), pp. 119-136

#
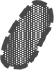


#
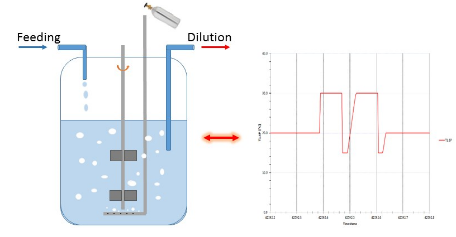
Strategy #13: Combined chemostat & temperature fluctuations

Ghil Jona & Dikla Levi

Department of Life Sciences Core Facilities, Weizmann Institute of Science, Rehovot, Israel

Description: Our *E. coli* strain has undergone *in-vivo evolution* at 20C using a chemostat approach combined with four cycles of temperature fluctuations (once a week). Each temperature fluctuation cycle was comprised of two relaxing growth phases (each of 1.5-2 hours at 30C), followed by two 15’ cold shock cycles at 15C, before returning to the evolution temperature (20C). For the first 3.5 weeks, the bacteria were grown at a dilution rate of 0.23 /hr. Then, when we observed an increase in the yield, the dilution was increased to 0.46 /hr for the remainder of the study.

Rationale: In vivo evolution using chemostat at quasi-exponential rate selects for cells adapted to grow faster than their neighboring cells in the limiting conditions (cold temperature in this study). Using this strategy we specifically select for faster growers and avoid the indirect selection of cells that may have a shorter exit from stationary, or more stable when reach stationary. Combining this strategy with several cycles of growth in relaxing followed by stressing conditions should in principle help enrich for cells that are more flexible and versatile: hence bacteria that can cope both with changes in the growth conditions, and still grow faster than the rest.

Materials and Methods: 1ml O.N. culture was used to inoculate a DASBox mini-bioreactor system containing 100 ml LB media supplemented with Kanamycin (50mg/lit). The bacteria were grown in batch mode in the bioreactor until reached saturation (~25hrs) and then shifted to chemostat mode using the same media at a dilution rate that kept a constant 3 hrs/doubling. Every 7 days the culture has undergone two consecutive cycles of temperature shifts that were as follows: a gradual increase of temperature in the bioreactor to 30C (1 C/min), followed by 1.5-2 hrs incubation at 30C (relaxing conditions), followed by a fast 15 min. cold shock cycle (15C), and gradual increase of temperature as above (see figure below). After 3.5 weeks, the OD of the culture increased, and we increased the dilution rate to 1.5 hrs/doubling for the next 3 weeks.


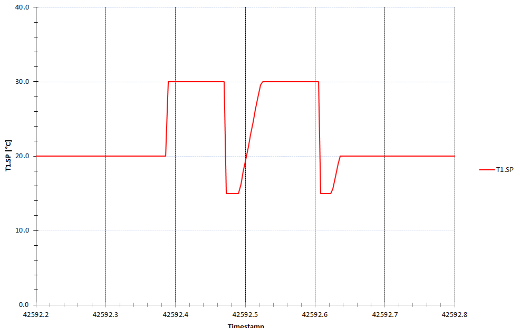


#
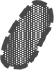

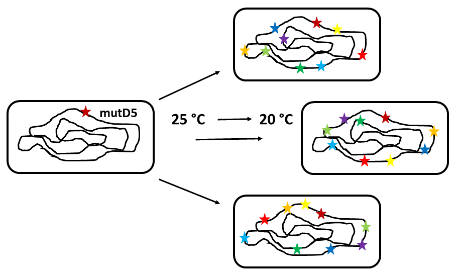
Strategy #14: Hypermutation evolution

Bálint Csörgő and Csaba Pál

Institute of Biochemistry, Biological Research Centre of the Hungarian Academy of Sciences, Szeged, Hungary

Description: We engineered the mutD5 mutator allele of *dnaQ*, which has been shown to have a mutation rate 1000-times greater than the wild-type level (1). We then used serial batch selection for adapting this strain for growth at cold temperature in two steps, growing at 25°C for an estimated 170 generations, followed by another estimated 170 generations of growth at 20°C.

Rationale: Our selected strategy for evolving *E.coli* for growth at 20°C was based on the rapid capability of mutator bacteria in adapting to new environments (2). Although not advantageous in the long-term, a mutator phenotype can help accelerate the accumulation of beneficial mutations (3).

Materials and methods: We generated the mutator allele in our barcoded strain of *E. coli* using single-stranded DNA (ssDNA)-mediated, λ-Red recombinase-induced allele replacement (recombineering) (4). Specifically, a C -> T mutation was introduced at position 236110 on the genome (within the *dnaQ* gene) resulting in a T15I mutation of the encoded enzyme. A ssDNA oligonucleotide (oligo) carrying the desired mutation was designed using the MODEST web tool (5) with the following name and sequence: dnaQ_T15I, 5’- T*C*TGGTTCATACCGGTGaTTTCGGTATCGAGAACGATCTGGCGTGTAATTGCAGTGCTCATAGCGGTCATTTATGTCAGACTTGTCGT*T*T, where asterisks indicate phosphorothioate bonds. Recombineering was then performed using the pORTMAGE-2 plasmid described previously (6). Briefly, the production of λ-Red Beta enzyme was induced by shifting the temperature of a culture of pORTMAGE-2 carrying barcoded *E. coli* cells to 42°C for 15 minutes. The cells were then electroporated with the dnaQ_t15I oligo and recovered overnight at 30°C. The culture was plated on LB + kanamycin (25 μg/ml) plates from which individual colonies were analyzed for carrying the desired mutation. Colony PCRs were performed using the dnaQ-HRM-Fw (5’ GCAATTACACGCCAGATCGTTC) and dnaQ-HRM-Rev (5’ CCACTTCAACGGCACCAATCTC) primers. The resulting fragments were then analyzed using High Resolution Melting analysis with the Luminaris Color HRM qPCR kit (Thermo Fisher) on a BioRad C1000 Touch thermal cycler. Mutation-carrying colonies were confirmed using Sanger sequencing. The cells were cured of the pORTMAGE-2 plasmid and a rifampicin-resistance mutation rate assay (7) was then performed to verify the mutator phenotype. Adaptation to 20°C started with the strain carrying the mutD5 allele. 10 μl of a starting culture (in LB + kanamycin) was transferred to 10 ml fresh LB and grown at 25°C for 24 hours. This was repeated 13 times, after which the culture was plated on LB + kanamycin plates. After 48 hours of growth at 25°C, the largest colony was chosen to inoculate a new starter culture in LB and grown at 20°C for 24 hours. 10 μl of this was transferred to 10 ml fresh LB media and grown once again at 20°C for 24 hours. 13 total transfers were performed at 20°C, with the final culture plated onLB + kanamycin plates. After 48 hours of growth at 20°C, the largest colony was chosen as the final adapted line. Thus, the total adaptation to low temperature occurred for an estimated 340 generations.

1. Fijalkowska IJ, Schaaper RM (1996) Mutants in the Exo I motif of Escherichia coli dnaQ: defective proofreading and inviability due to error catastrophe. *Proc Natl Acad Sci* 93(7):2856–2861.

2. Taddei F, et al. (1997) Role of mutator alleles in adaptive evolution. *Nature* 387(6634):700–702.

3. Giraud A, et al. (2001) Costs and benefits of high mutation rates: adaptive evolution of bacteria in the mouse gut. *Science* 291(5513):2606–2608.

4. Court DL, Sawitzke JA, Thomason LC (2002) Genetic Engineering Using Homologous Recombination1. *Annu Rev Genet* 36(1):361–388.

5. Bonde MT, et al. (2014) MODEST: a web-based design tool for oligonucleotide-mediated genome engineering and recombineering. *Nucleic Acids Res*:gku428.

6. Nyerges Á, et al. (2016) A highly precise and portable genome engineering method allows comparison of mutational effects across bacterial species. *Proc Natl Acad Sci*:201520040.

7. Foster PL (2006) Methods for Determining Spontaneous Mutation Rates. *Methods Enzymol* 409:195–213.

#
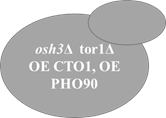
Strategy #15: Delete and prosper

Dmitry Zabezhinsky and Jeffrey Gerst

Department of Molecular Genetics, Weizmann Institute of Science, Rehovot, Israel

Description: Deletion of genes that confer cold sensitivity, and over expressing genes that confer cold resistance.

Rationale: By modifying genes that are cold-related, the yeast cell will have the advantouge of growing in cold, since genes that confer sensitivity to cold were deleted, and genes that confer resistance to cold are over expressed.

Materials and methods

A manual curated search was made in SGD database using the keyword “Cold sensitivity”. Candidates genes (*OSH3*, *INP51*,*DET1* and *RAS2*) were chosen to be deleted based on highist number of publications indicating that a deletion confer cold resistance.

First, the single deletions were made by replacing the relevant gene with a selectable marker^1^ using homologous recombination . Growth was checked via spot assay at 26 and 15C. The best single deletion mutants were subjected to aditional round of deletion with additional curated genes.

Double mutants were checked again using spot assay to find the best cold resistant strain.

The keyword “Cold sensitivity” was mined again in SGD database, however now for over expression phenotype. Candidate genes (*CTO1* and *PHO90*) were integrated into the yeast genome under the regulation of the TEF3 strong promoter using pFA6-TEF3 plamids^2^.

The iterative OE mutants strains were checked for cold resistance using spot assay and the best candidate mutant strain (osh3Δ,tor1Δ,OE CTO1, OE PHO90) was submitted to Yitzhak Pilpel lab for the competetetion.

1. Yeast. 2004 Aug;21(11):947-62.
2. Yeast. 1998 Jul;14(10):953-61.

#
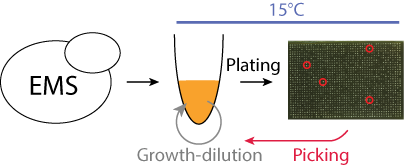
Strategy #16: Chemical mutagenesis

Joseph M Georgeson, Ehud Sass, Emmanuel D Levy

Weizmann Institute of Science, Department of Structural Biology, Rehovot, Israel.

Brief description
The sample was submitted to EMS-induced mutagenesis. The resulting population was grown at 15°C with dilution every 96 hours for a total of 7 weeks. Twice during these growth cycles, the culture was printed on ten agar plates with a 1536-format pintool, and the largest colonies were pooled together to continue growing.

Rationale
Strains having accumulated EMS-induced mutations beneficial for growth at 15°C were expected to take over the population. Pining to high density agar plates and subsequent picking of large colonies was expected to speed-up the process.

Materials and Methods

Ethyl methanesulfonate (EMS) mutagenesis was used based on a previously published protocol [(Winston 2008)](https://paperpile.com/c/QBELgx/BvDs). From the provided sample, a 5 mL culture was grown in YPD + hygromycin (300 µg/mL) overnight at 30°C. Cell density was determined and 2x108 cells were prepared for EMS mutagenesis by pelleting, washing twice with 1.0 mL of sterile water, and resuspending in 1.5 mL 0.1 M PBS, pH 7.0. Half of the cells were kept as a control, and 50 µL EMS was added to the other half, which was briefly vortexed and placed on a rotating platform at room temperature. After 1 hr, 0.2 mL of both the treated and non-treated cell samples were added to 8 mL 5% sodium thiosulfate for 5 minutes to inactivate EMS. The samples were pelleted, washed twice with 1.0 mL of sterile water, resuspended in 3.0 mL YPD, and incubated for 3 hrs at 30°C before plating. Plating of serial dilutions on YPD + hygromycin plates showed that approximately 50% of cells were killed during EMS treatment, and plating on SD + canavanine (50 µg/mL) plates showed that treated cells had a 10-fold increase in resistance to canavanine compared to the control indicating significant mutagenesis rate. The remaining non-plated culture was added to liquid YPD + hygromycin for a final volume of 10mL in a 50 mL falcon tube with air-permeable cap, and allowed to grow at 15°C. Cell density was measured and adjusted to a cell density of ~5x106 every 96 hours. After 3 weeks, a 1,536-floating pin tool was used to transfer cells to ten YPD + hygromycin plates. The sample was prepared for pinning by diluting to 2.5x105 cells/mL and passing through a 35 µm cell strainer before transferring 35 mL to a reservoir for pinning. This cell density was calibrated to result in no growth for ~5% of positions in the plate, an indication that a pin transferred only a single or a few cells. Following growth at 15°C for 1 week, the largest ~50 colonies from each plate were pooled, grown in liquid YPD + hygromycin at 15°C for 2 weeks as previously described, after which the pinning process was repeated once more. Colonies from the second pinning were significantly more homogenous than the first. The pooled culture from the second pinning-selection was grown in liquid YPD + hygromycin for another 2 weeks. A random colony from the pool was chosen to submissition.

Reference

[Winston, Fred. 2008. “EMS and UV Mutagenesis in Yeast.” Current Protocols in Molecular Biology / Edited by Frederick M. Ausubel ... [et Al.] Chapter 13 (April): Unit 13.3B.](http://paperpile.com/b/QBELgx/BvDs)

#

#
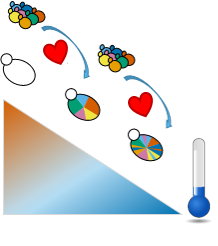
Strategy #17: Breeding with natural variation

Alex N. Nguyen Ba, Michael M. Desai

Department of Organismic and Evolutionary Biology, Harvard University, Cambridge, MA 02138, USA; FAS Center for Systems Biology, Harvard University, Cambridge, MA 02138, USA.

Short description and rationale: The chosen evolutionary strategy selects on standing genetic variation in wild yeast populations (1) while maintaining the presence of the barcode. We cycled between rounds of outbreeding and rounds of selection. To accelerate the breeding process, the selection temperature was continuously decreased over breeding cycles as the strain adapted to low temperature.

Recombination selects on existing variants, allowing the purge of deleterious mutations from the barcoded strain, while incorporating beneficial mutations. There are thousands of mutations in wild strains, many of which are already better adapted to lower temperature than the laboratory strain.

Materials and Methods: To make crossing and selection to the SGRP amenable in high-throughput, we first modified the starting strain Y7.3 to make it compatible with synthetic genetic array methodology (2). This technology requires at the minimum these three systems: 1) diploid selection, 2) mating-type specific selection, 3) diploid killing following sporulation. Because the SGRP collection is G418 and Hygromycin B resistant (markers at URA3 and HO respectively), we first modified the hygromycin B marker in the starting evolthon strain Y7.3 to the nourseothricin resistance marker using standard gene replacement (YAN502). We used the MATa SGRP collection as the outbreeding population, and therefore we swapped the mating type of our strain to MATalpha (YAN507), and introduced at the can1 locus the URA3 gene under the control of the mating-type specific promoter of Ste3 (YAN514), which is only active in MATalpha cells. The disruption of the CAN1 gene also allows specific killing of diploids using canavanine.

The SGRP MATa collection was grown as a pool overnight in YPD at room temperature, and mated to YAN514. Diploid cells were then selected using CloNAT (100ug/mL), Hygromycin B (300ug/mL) and G418 (200ug/mL). After the initial cycle of diversification, the cells were propagated at decreasing temperature at a 1/1000 dilution cycle. Our goal was to periodically outcross the selected pool of cells. To restart this process, the cells were sporulated in SPO media (1% KoAc, 10ug/mL Uracil) for three to five days, or until 90%+ of the population had sporulated. After a brief zymolyase treatment, and vigorous bead beating to separate the meiotic progenies, the population was passaged to minimal media (YNB without ammonium sulfate, 1g/L monosodium glutamate) with amino acids but lacking uracil, containing canavanine (50ug/mL) and CloNAT. Grown haploid MATalpha cells were then crossed back to the SGRP collection as previously outlined. We continued the cycle of growth and outcrossing for a total of 6 crosses and a total of 150 generations of growth at decreasing temperature.

References

1.Cubillos, F. A., Louis, E. J. & Liti, G. Generation of a large set of genetically tractable haploid and diploid Saccharomyces strains. FEMS Yeast Res. 9, 1217–1225 (2009).

2.Tong, A. H. Y. & Boone, C. Synthetic Genetic Array Analysis in Saccharomyces cerevisiae. in Yeast Protocol (ed. Xiao, W.) 171–191 (Humana Press, 2006).

#

#
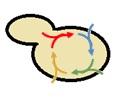
Strategy #18: Simply Metabolism

Alice Flint^1^, Andras Solt^1^, Prashant Kumar^1^, Viridiana Olin-Sandoval^1,4^, Markus Ralser^1,2,3^.

^1^Department of Biochemistry, University of Cambridge, UK.

^2^The Molecular Biology of Metabolism laboratory, The Francis Crick Institute.

^3^Department of Biochemistry, Charitè University Medicine, Berlin, Germany.

^4^Department of Nutrition Physiology, Instituto Nacional de Ciencias Medicas y Nutricion Salvador Zubiran, Mexico City, Mexico.

Description:

Metabolic deficiencies of original strain (Y18) were repaired by mating with the prototrophic haploid *S. cerevisiae* YSBN3. Then, the diploid strain was evolved using chemical mutagenesis with EMS followed by growth at 15°C, alone and in competition with original strain. The final winner was picked from a competition between the fastest colonies.

Rationale:

The idea of the simple Metabolism strategy was to repair the metabolic deficiencies (auxotrophies) in the background strain, before evolving it at cold temperature. Repairing the auxotrophic markers will restore metabolic capacities gaining a head start on the competition. Diploidy recapitulates gene duplication that will enhance the genetic pool for mutation and thus, will give the strain plasticity to adapt to new environmental conditions.

Materials and Methods:

Original strain (*MAT*a) and YSBN3 (*MAT* alpha; ho::loxP-*TEF1*p-ble-*TEF1*t-loxP; ura3-52::*URA3*) *S. cerevisiae* strains were mated by streaking in a perpendicular pattern onto YPD (10 g/L yeast extract, 20 g/L peptone, 20 g/L glucose) agar plates, and grown for 48 h at 30 °C. The crossover point was streaked on SM agar plates (6.8 g/L Yeast Nitrogen Base, 2% glucose) with the antibiotics hygromycin (300 µg/mL) and phleomycin (7.5 µg/mL), and grown for 48 hours at 30 °C to select diploid strains (Y18:YSBN3).

Y18 and selected diploid strains were mutagenised using ethyl methanesulphonate (EMS). Strains were grown overnight in YPD at 30 °C, 140 rpm shaking. 2 mL of each culture was then diluted to an OD_600_ of 2 AU in sterile water and centrifuged at 21,000 rcf for 1 min. The pellet was washed with water and resuspended in 1.5 mL 0.1 M phosphate buffer pH 7.4 and 3% EMS. The culture was incubated at 30°C, shaking at 140 rpm. 300 µL of culture were taken at 30 and 60 min and centrifuged at 21,000 rcf for 5 min. The pellet was washed in 1.5 mL of 5% sodium thiosulphate three times to quench the mutagenesis (Winston, 2008). The resulting culture was used to inoculate 25 mL of YPD media and allowed to recover at 30 °C, 140 rpm shaking for 48 h.

The resulting eight strains (two from each timepoint, for both Y18 and Y18:YSBN3) were diluted to 0.02 AU OD_600_ every 24 h and grown at 15 °C for four passages. Each culture was plated on YPD agar, and 4 colonies from each plate were selected for growth curve analysis. Colonies were grown in 2 mL of YPD media overnight at 15 °C, 140 rpm shaking and diluted to an OD_600_ 0.02 into 200 µL YPD media, in an optically clear 96 well plate. Each colony was inoculated in triplicates. Growth curves were measured using a Tecan Infinite M200 Pro, taking OD_600_ readings at 10 min intervals for 48 h at 15 °C. Growth curves were analysed using a spline model from the Grofit package in R (Kahm et al, 2010).

The 3 ‘winning’ strains were pooled and passaged for three generations at 15 °C as described earlier, in a 1:1 starting dilution with Y18, to evolve the strains under competition. The resulting culture was plated on SM agar with hygromycin to confirm ploidy. The growth curves of four SM colonies were recorded. The fastest three strains were pooled and subjected to a further round of mutagenesis, as detailed above with an additional 75 min time point. Each culture was passaged for four generations at 15°C, again as above. The growth curve for each culture were assayed in the presence Y18 in a 1:1, 1:10 and 1:25 ratio as described above. The three fastest growing strains were plated on SM agar with phleomycin (7.5 µg/mL) and hygromycin. Four colonies were picked from each plate, and their growth characteristics assayed at 15 °C. The fastest growing colony was selected as the final winner and streaked on SM media with phleomycin and hygromycin to confirm parental characteristics.

Kahm M, Hasenbrink G, Lichtenberg-Fraté H, Ludwig J, Kschischo M (2010) grofit: Fitting Biological Growth Curves with R. J. Stat. Softw. 33(7): 1-21

Winston F (2008) EMS and UV Mutagenesis in Yeast. Curr Protoc Mol Biol. 82(1): 13.3B.1-13.3B.5

#
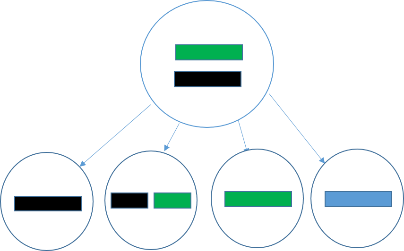
Strategy #19: Adaptive lab evolution with mating

Razi Zeidan, Iftach Nachman

Department of Biochemistry and Molecular Biology George S. Wise Faculty of Life Sciences, Tel Aviv University

Description: *S. cerevisiae* cells were mated with a cold-tolerant strain, and then back-crossed to the competition strain, with intermittent adaptation to 15˚C.

Rationale: Some wild strains have evolved for optimal growth at cold temperatures. Repeated back crossing to the lab strain can distill the relevant gene variants, if combined with selection cycles.

Materials and Methods:

To select the wild strain to be used, we ran a competition between the various wild strains that we expected to have higher fitness in relatively low temperatures. Two criteria that we considered were environment (e.g. cold countries) and ecology (e.g. cold fermentations).

The competition consisted of growing the yeast cultures in 15˚C overnight, and comparing the density via OD. To account for differences in cell size, we grew the yeast cultures beforehand until they reached saturation and then measured their OD. Thus, we were able to calculate the percentage of growth in 15˚C. Our winner was a strain used for Sake (where fermentation temperature is usually between 0˚C - 10˚C), which is essential for Japanese rice wine making.

In order to allow the formation of stable haploid strains, we knocked out the HO gene (which gives the yeast the ability to change its mating type) of the sake strain by replacing it with a G418-resistance cassette.


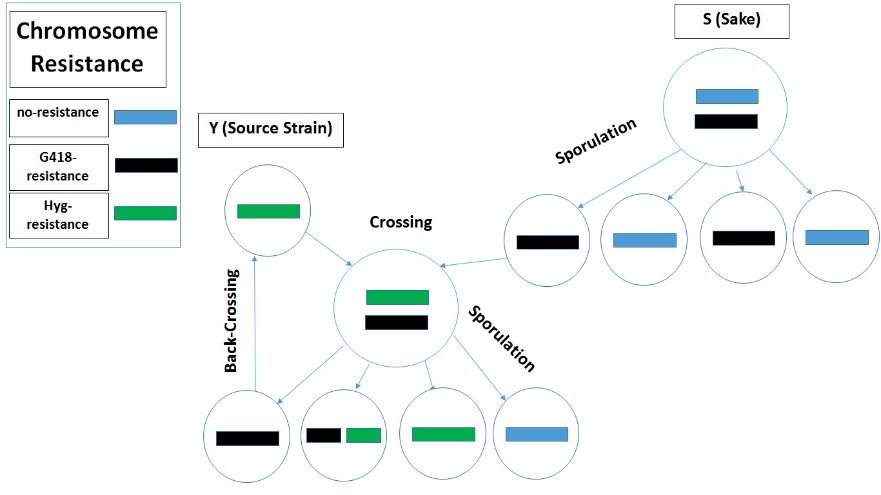
We then conducted one crossing and two back crossings with the source strain from three different generations (almost one month apart) that were grown in 15˚C over two months. After each cross or backcross, the resulting diploids were sporulated on SPM plates. For the aim of back-crossing, we had to select the spores that only had G418-resistance, in order to cross with the source strain which has Hyg-resistance. To do so, single colonies were grown on G418 plates, and then replica-plated onto G418+Hyg plates. For the next round, colonies that grew only on the G418 plates were chosen. These haploid strains were then grown in 15˚C, and those with the highest growth rates at 15˚C were used in the next round of back-crossing.

#
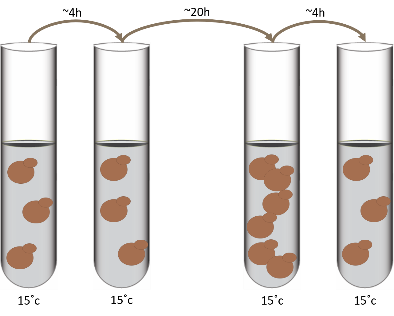
Strategy #20: *S. cerevisiae* Manual chemostat

Avihu H. Yona

Physics of Living Systems, Department of Physics, Massachusetts Institute of Technology, Cambridge, MA, 02139, USA

Description: *S. cerevisiae* strain was adapted to cold by diluting the cells twice a day.

Rationale: Aiming to reduce lag phase, cells were diluted twice a day, where the second time was couple of hours after the first dilution of the day.

Materials and Methods: Daily dilution of 1:100 was made twice a day into 1ml of fresh YPD at 20oC (on a horizontal shaker). Each day the second dilution was done between 2-8h after the first dilution.

#
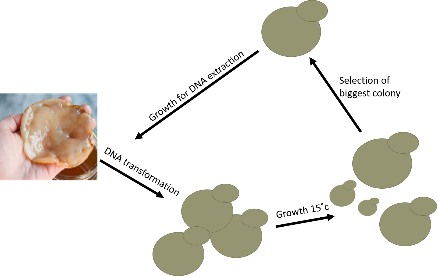


# Strategy #21: Foodie-evolution

Shira Nouriel, Michael Klutstein, Ronen Hazan

Faculty of Dental Medicine, The Hebrew University of Jerusalem, Jerusalem 9112001, Israel

Description: *S. cerevisiae* cells were adapted to growth in a constant temperature stress of 15˚C by serial transformation-selection cycles of the *S. cerevisiae* population. Repeated cycles of transformation (7 cycles of transformation were done) of DNA from Kombucha and sourdough yeasts into the original strain, growth at 15˚C, and selection of the biggest colony for the next cycle were performed.

Rationale: Yeast strains which were isolated form sourdough and Kombucha must be selected for growth and survival at cold temperatures, since they are kept for many moths in the refrigerator. We hypothesized that they could contribute cold- resistance genes, or cold resistance version of genes.

Materials and Methods:

A culture of Kombucha Saccharomyces cerevisiae purchased from happyherbalist.com (https://www.happyherbalist.com/kombucha-mushroom-culture-saccharomyces-boulardii/ ) and a culture of lab-grown sourdough Saccharomyces cerevisiae (from S288C strain), both kept in a refrigerator (4 ˚c ) for 6 months were mixed, and genomic DNA was extracted. DNA was fragmented by sonication, and blunted by a fill-in reaction with T4 DNA polymerase (NEB).

*S. cerevisiae* cells from the organizers were grown in 1.2ml of YPD media (10g/L yeast extract, 20g/L peptone, 20g/L Glucose) in a flask at 15˚X. Upon reaching stationary phase (~48 hours) cells were diluting 1:120 into fresh YPD media. Cells were transformed with the DNA from the food yeasts. After each transformation, the transformed cells were plated on YPD-agar plates, and incubated in 15˚C for a week. Following incubation, the biggest colony was taken and the process was repeated. In total, 7 transformation rounds were performed. The biggest colony in the last round was taken for the Evolthon challenge.

# *
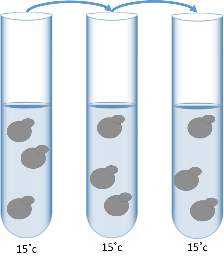
*Strategy #22: *S. cerevisiae* Daily dilution

Sivan Kaminski Strauss, Orna Dahan, Yitzhak Pilpel

Department of Molecular Genetics, Weizmann Institute of Science, Rehovot, Israel

Description: *S. cerevisiae* cells were adapted to growth in a constant temperature stress of 15˚C by daily diluting the evolving *S. cerevisiae* population.

Rationale: This strain serves as a base line for evolution under low temperature. By design its evolution was under simplest possible setup that only exposes cells to low temperature.

Materials and Methods:

*S. cerevisiae* cells were grown 1.2ml of YPD media (10g/L yeast extract, 20g/L peptone, 20g/L glucose) in a 24-well plate under constant shaking of ~800rpm at 15˚C. Upon reaching stationary phase (~48 hours) cells were diluting 1:120 into fresh YPD media. Evolution was done for ~130 generation in 4 repetitions.

At the end of the evolution, samples from each of the evolving populations were plated on YPD-agar plates. Single colonies were picked randomly for growth experiment in a robotic system. Each colony was incubated in liquid YPD media and grown for 48-hours at 15˚C while shaking at ~800rpm, OD600 was measured at 30min intervals, and the best performing colony was chosen as the participant strains for Evolthon Challenge.

#

#
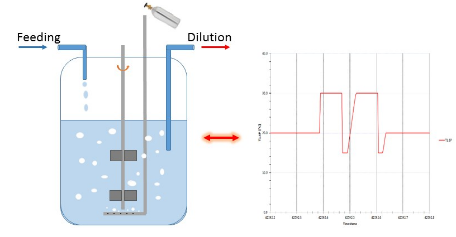
Strategy #23: Combined chemostat & temperature fluctuations

Ghil Jona & Dikla Levi

Department of Life Sciences Core Facilities, Weizmann Institute of Science, Rehovot, Israel

Description: Our *yeast* cells have undergone *in-vivo evolution* at 15C using a chemostat approach combined with two cycles of temperature fluctuations (weeks 4 & 6 of the evolution). Each temperature fluctuation cycle was comprised of two relaxing growth phases (each of 3 hours at 25C), followed by two 15’ cold shock cycles at 12C, before returning to the evolution temperature (15C). For the first 7 weeks, the yeast cells were grown at a dilution rate of 0.14 /hr. The last week of the experiment, we increased the dilution rate to 0.18/hr.

Rationale: In vivo evolution using chemostat at quasi-exponential rate selects for cells adapted to grow faster than their neighboring cells in the limiting conditions (cold temperature in this study). Using this strategy we specifically select for faster growers and avoid the indirect selection of cells that may have a shorter exit from stationary, or more stable when reach stationary. Combining this strategy with several cycles of growth in relaxing followed by stressing conditions should in principle help enrich for cells that are more flexible and versatile: hence bacteria that can cope both with changes in the growth conditions, and still grow faster than the rest.


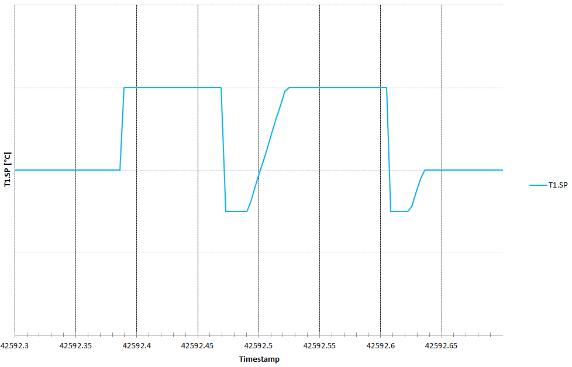
Materials and Methods: 2.5ml O.N. culture was used to inoculate a 1 lit BF110 bioreactor (New-Brunswick Scientific – Eppendorf) containing 500ml YPD media supplemented with Hygromycin B (100 mg/lit). The yeast cells were grown in batch mode for 15 hours and then shifted to chemostat mode using the same media that was fed and diluted at a rate that retained a growth rate of 7 hrs/doubling. After four weeks and 6 weeks in chemostat mode, the culture was subjected to two consecutive cycles of temperature shift that were as follows. A gradual increase of temperature in the bioreactor from 15C to 25C (over 20 minutes), followed by 3 hrs incubation at 25C (relaxing conditions), followed by a fast 15 min. cold shock cycle (12C), and finally a gradual increase of the temperature as above (see figure below). For the last week of the experiment, we increased the dilution rate to 5.5 hrs/doubling.

#
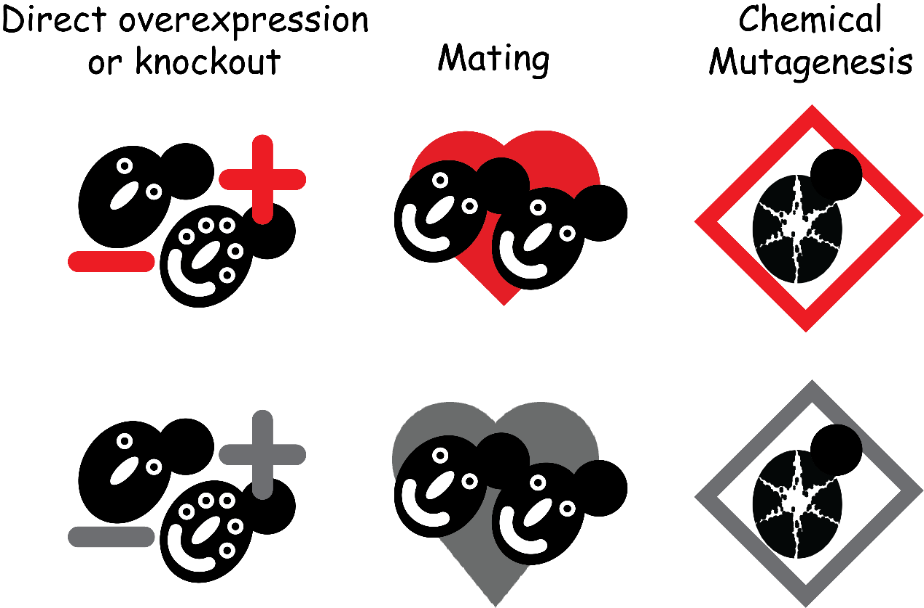
Strategy #24: Engineering of cold response genes using CRISPR/Cas9

Marc Rübsam, Bede Busby, Aaron N. Brooks, Bianca P. Hennig, William F. Mueller, Daniel Schraivogel, Sibylle C. Vonesch, Lars M. Steinmetz

European Molecular Biology Laboratory (EMBL), Genome Biology Unit, 69117, Heidelberg, Germany.

Description: Using CRISPR/Cas9 we attempted to generate loss-of-function alleles of genes conferring cold sensitivity and introduce short, highly active, synthetic promoters in front of genes whose overexpression has been linked to decreased cold sensitivity.

Rationale: These genes have a known function in mediating the cold-stress response and we assumed their perturbation in the indicated direction would improve cold tolerance.

Materials and Methods: Seven genes with phenotype “cold sensitivity: decreased” and mutant type “null”, “reduction of function”, or “conditional” were chosen for loss-of-function (YDR051C, YIL002C, YNL106C, YKR048C, YHR073W, YNL098C, YJR066W), while four candidates with phenotype “cold sensitivity: decreased” and mutant type “overexpression” were selected for overexpression (YCR015C, YKR097W, YJL198W and YDR007W) (annotations from [SGD](https://www.yeastgenome.org/)). Knockout sgRNAs were designed with ECRISP (1), using *S.cerevisiae* R64-1-1 and options strict, any 5’ preceding base, and 50 results per exon. Designs were ranked according to specificity, accessibility (custom score based on own unpublished yeast ATAC-seq data), efficacy and annotation, and the 2-5 highest-scoring sgRNAs per gene synthesized with overlaps for Gibson assembly (5’tcggcgttcgaaacttctccgcagtgaaagataaatgatcNNNNNNNNNNNNNNNNNNNNgttttagagctagaaatagcaagttaaaataaggctagtc 3’, where N denotes the 20bp specific to each sgRNA). sgRNAs were made double stranded using a reverse complementary oligo (longer 3’ extender) in a Klenow extension reaction, assembled into the p427TEF_Cas9_guide_AatII plasmid (NEB Gibson Assembly Master Mix) and transformed into NEB 10-beta cells. 20/22 sgRNAs (representing all 7 genes) were used in subsequent experiments. Each plasmid was transformed separately into barcoded strain via electroporation and transformed cells were plated on YPAD plates (10g yeast extract, 20g peptone, 20g glucose, 40mg adenine sulfate) containing G418 (400µg/mL).

For overexpression we designed a fragment encoding a short synthetic promoter (triple UAS core) based on (2). We determined transcription start sites (TSS) for our candidate genes using 5’P sequencing data (3, own unpublished data) of the strain BY4742, and designed sgRNAs targeting approximately 40bp upstream of the TSS using [Benchling](https://benchling.com). We also designed an sgRNA to knock out an upstream repressive element whose deletion results in a 8-fold upregulation of PCK1 (4). Overexpression sgRNAs were cloned into the [Ellis lab CRISPR system](https://benchling.com/pub/ellis-crispr-tools), which allows targeting of multiple loci simultaneously. Donors were obtained by amplifying the triple UAS core oligo with primers containing 50bp overlaps specific to the insertion sites (donors for overexpression). The triple UAS core oligo was made double stranded by extension from a short complementary oligo (triple_UAS_core_revC), homology overlaps were added by amplifying the double stranded fragment with the respective set of primers for each locus for 10 cycles and the resulting donor fragment was purified using ethanol precipitation. The donor for knockout of the PCK1 repressive element (donor PCK1 rep. element KO) was designed as two partially complementary fragments with 55bp of homology to the genomic region immediately up- and downstream of the repressive element. The fragment was made double stranded in a 1-cycle extension reaction using 2µL of each oligo (100µM) and ethanol purified. pWS174 and sgRNA plasmids were linearized and gel purified (Qiaquick Gel Purification kit). For electroporation into yeast, sgRNA cassettes were pooled and mixed with linearized plasmid and 5µg of each donor. Transformed cells were plated on YPAD plates containing clonNAT (200µg/mL). In addition to the barcoded strain, we transformed strain 2.3 (previously targeted with sgRNA YKR048C_12_750 targeting NAP1), which had shown slightly improved growth at 15°C, as well as a pool of five (non-validated) clones per sgRNA from the targeted knockout transformation, to allow for identifying beneficial interactions between different modifications. Growth performance of individual colonies at 15°C was assessed by measuring OD595 every ~4h for 48h using a TECAN Genios. Between measurements, 96-well plates were incubated at 15°C and 150rpm. The clone showing the best growth performance at 15°C was selected.

1. Heigwer, F. , Kerr, G. & Boutros, M. E-CRISP: fast CRISPR target site identification. Nat. Methods 11, 122-123 (2014).
2. Redden, H. & Alper, H. S. The development and characterization of synthetic minimal yeast promoters. Nat. Communications 6, (2015).
3. Pelechano, V., Wei, W., & Steinmetz, L. M. Widespread Co-translational RNA Decay Reveals Ribosome Dynamics. Cell 161, 1400 – 1412 (2015).
4. Mercado, J. J. & Gancedo, J. M. Regulatory regions in the yeast FBP1 and PCK1 genes. FEBS Letters 311, 110-114 (1992).

**(See oligo sequences attached to this strategy in** [**Evolthon site**](https://evolthon2016.wixsite.com/home)**)**


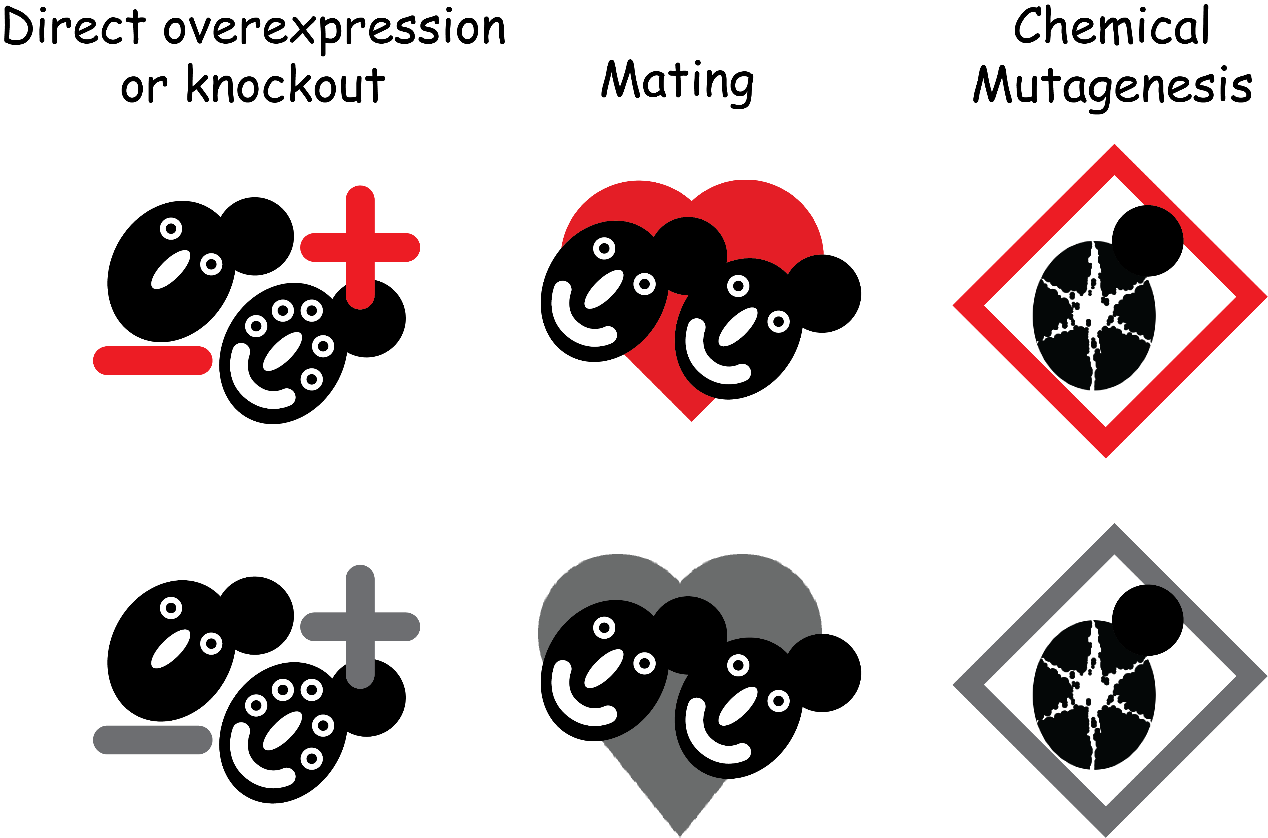
Strategy #25: Cycles of random mutagenesis with selection

Marc Rübsam, Bede Busby, Aaron N. Brooks, Bianca P. Hennig, William F. Mueller, Daniel Schraivogel, Sibylle C. Vonesch, Lars M. Steinmetz

European Molecular Biology Laboratory (EMBL), Genome Biology Unit, 69117, Heidelberg, Germany.

Description:

We evolved strains through alternating cycles of random chemical or UV mutagenesis and selection of best performers. After each round of mutagenesis the 10% best performing strains were identified by phenotyping at 15°C and used in two separate pools (best half, other half) for the next round of mutagenesis.

Rationale: We reasoned that repeated cycles of random mutagenesis followed by selection of the best growers at 15°C could result in a strain with superior growth performance at cold temperatures faster than by adaptation.

Materials and Methods: A single colony of the competition strain was inoculated in YPAD medium (10g yeast extract, 20g peptone, 20g glucose, 40mg adenine sulfate) and incubated overnight at 30°C, 150 rpm. The culture was diluted to an OD600 = 0.2 and grown at 30°C to an OD600 = 1.6. For EMS mutagenesis, 1 ml of this culture was harvested and washed twice with 20 ml deionized water. The pellet was resuspended in 1 ml 0.1 M sodium phosphate pH 7.0. Aliquots of washed yeast cells were treated with 30 µl EMS (SIGMA-ALDRICH, 1.206 g/ml) and incubated for 1 hour at 30°C on a turning wheel. The cells were pelleted and washed three times with 5 % sodium thiosulfate to inactivate and completely remove the EMS. Washed cells were resuspended in 1 ml deionized water and 100µl of a 1:100 dilution were plated on YPAD–agar plates containing Hygromycin (200µg/ml).

For UV mutagenesis, 50mL cells were pelleted and spread on YPAD+Hygromycin plates. Cells were irradiated in a Spectrolinker XL-1500 UV chamber (SPECTROLINE) for 15s and recovered at 30°C for 2 days. Growth performance of individual colonies at 15°C was assessed by measuring OD595 every ~4h for 48h using a TECAN Genios. Between measurements, 96-well plates were incubated at 15°C and 150rpm. As recovery at 30°C and subsequent phenotyping at 15°C was faster than direct selection at 15°C and resulted in comparable numbers of superior colonies we used this strategy for identification of candidates.

Cells then underwent five cycles of mutagenesis, recovery and phenotyping. In each cycle, cells were treated in parallel with EMS and UV. In cycle 1 we used the competition base strain. In subsequent cycles, the overall best performing 10% of colonies was identified via phenotyping and two pools of the best and second best 5% were made for the next round of mutagenesis. The best colonies from previous rounds were always included in the phenotyping of the next cycle. In the end the clone with the overall best performance was selected, irrespective of which cycle it derived from.

#
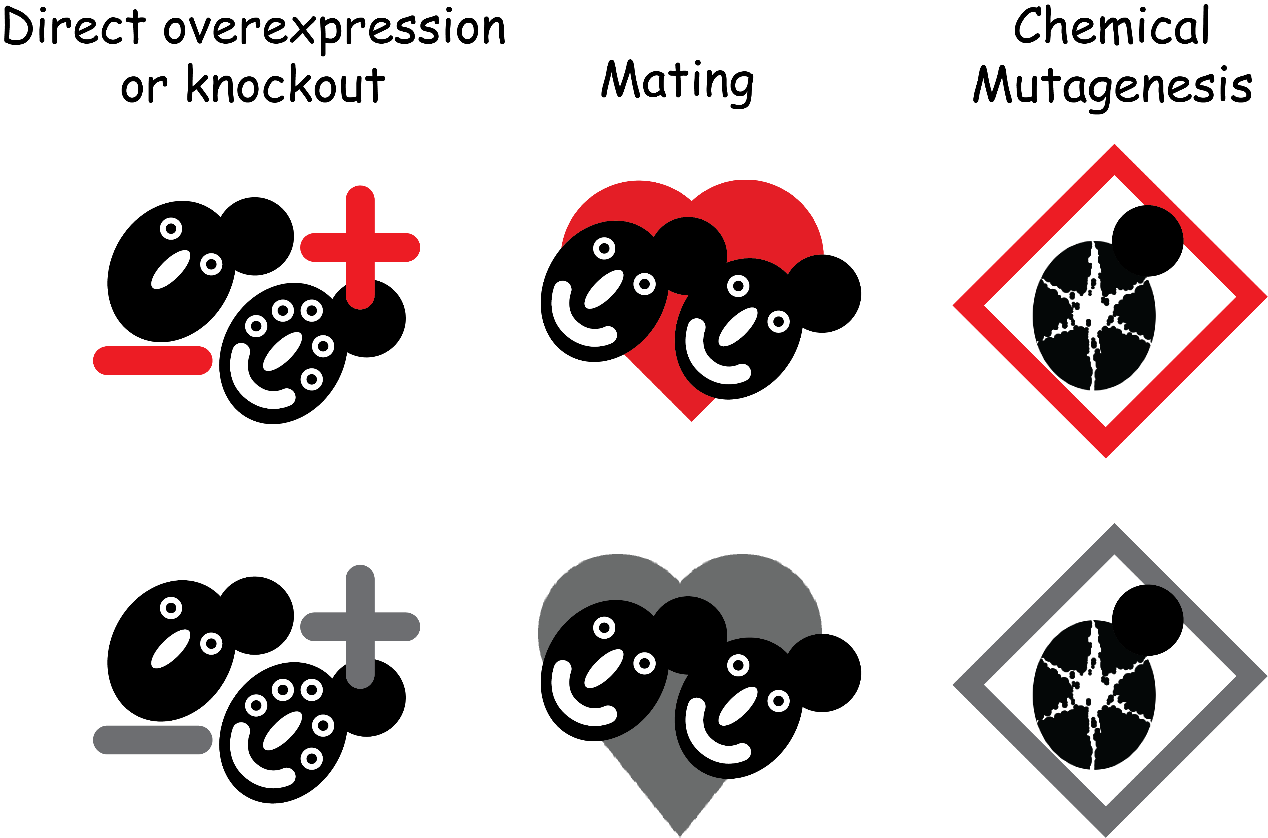
Strategy #26: Mating

Marc Rübsam, Bede Busby, Aaron N. Brooks, Bianca P. Hennig, William F. Mueller, Daniel Schraivogel, Sibylle C. Vonesch, Lars M. Steinmetz

European Molecular Biology Laboratory (EMBL), Genome Biology Unit, 69117, Heidelberg, Germany.

Description: We mated our competition strain with a wild type strain with superior growth performance at 15°C, followed by a round of random mating (two rounds of interbreeding total).

Rationale: We hypothesized that introducing genetic material from a cold tolerant strain via mating and meiotic recombination should improve cold tolerance of the resulting spores.

Materials and Methods: YJM1244, a sequenced wild isolate from Indonesia belonging to the wine subgroup (1) was selected based on its superior growth performance at 15°C (as reported in the paper). The HO locus in YJM1244 was replaced with a nourseothricin resistance cassette to be able to generate stable MATα haploids. The NatR cassette was amplified from plasmid p183 using primers that add 55bp overlaps homologous to the genomic region surrounding AJU86282.1 (CP004702.1, start = 36085, end = 37845), the YJM1244 gene equivalent to S288c HO (YDL227C). YJM1244 was transformed with 5µg of the deletion cassette and cells were plated on YPAD-agar plates (10g yeast extract, 20g peptone, 20g glucose, 40mg adenine sulfate) containing clonNAT (200µg/mL). Insertion was confirmed by PCR and successful transformants were sporulated in medium 1 (Materials) at 25°C for 5 days. Tetrads were dissected on a Singer MSM 400 microscope and the mating type was determined via PCR carried out as described in 2 to select MATα HOΔ (NAT) haploid cells.

An advanced intercross design (modified from 3) was used for mating. Two replicates of stable MATα haploids derived from YJM1244 were mated with the competition base strain on YPAD for 1 day at 30°C, diploids selected on double selection media (YPD+HYG+NAT) for 2 days at 30°C and sporulated in 5 mL sporulation medium for 5 days at 25°C (set up in four different media, 1 to 4). The spores from the different media were combined and pelleted by centrifugation (3000g, 5 min), washed with deionized water, re-suspended in 1 mL 100mM Tris-HCl (pH 9.4), 10 mM DTT and incubated at 30°C (water bath) for 10 min. After incubation the cells were harvested by centrifugation (13000g, 1min) and re-suspended in 0.5mg/mL zymolyase 100T (AMSBIO) in 2.1 M sorbitol, 10mM sodium dihydrogen phosphate (pH 7.2) and incubated at 30°C for five hours in a water bath. The viable cells were again pelleted by centrifugation (13000g, 1 min), and resuspended in 300µl 1 mM DTT, 0.4% w/v SDS and sonicated in a Bioruptor Pico water bath sonicator (DIAGENODE) for 2 min (4 cycles, 30s sonication, 1 min break). After sonication, the volume was brought to 1mL and additional SDS was added to raise the final concentration to 1%. The cells were incubated at 37°C for 1 h followed by 10 min at 55°C. The viable haploid progeny were harvested (2 min, 13000g), resuspended in 100µl YPAD, spread on a YPAD plate and incubated at 30°C for 2 days of random mating. Cells were then washed off plates and spread on double selection (YPAD+HYG+NAT) plates to select for diploids. The resulting diploids were sporulated at 25°C for 5 days (all four media), from which 90 tetrads were dissected (from media 3 and 4 for biological replicate 1 and from medium 2 for replicate 2) and grown on YPAD plates for 2 days at 30°C. Mating type was determined as described above. Growth performance of individual spores at 15°C was assessed by measuring OD595 every ~4h for 48h using a TECAN Genios. Between measurements, 96-well plates were incubated at 15°C and 150rpm. The fastest growing Hyg resistant spore was selected for submission.

Sporulation Media (1 L):

Medium 1: 10g potassium acetate, 1g yeast extract, 0.1 g amino acid mixture (2g His, 10g Leu, 2g Lys, 2g Ura), 0.5g glucose

Medium 2: 10g potassium acetate, 0.1 g amino acid mixture, 25mg glutamate

Medium 3: 10g potassium acetate, 0.1 g amino acid mixture

Medium 4: 10g potassium acetate

1. Strope, P. K. et al. The 100-genomes strains, an S. cerevisiae resource that illuminates its natural phenotypic and genotypic variation and emergence as an opportunistic pathogen. Genome Research 25, 1-13 (2015).
2. Huxley, C., Green, E. D. & Dunham, I. Rapid Assessment of S. Cerevisiae Mating Type by PCR. Trends in Genetics 6, 236 (1990).
3. Roberts C. 2015 “Investigating the quantitative trait loci contributing to individual variation in drug response” Thesis, Victoria university of Wellington, New Zealand.

#
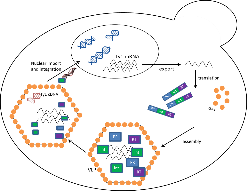
Strategy #27: Ty-induced evolution

Sivan Kaminski Strauss, Yitzhak Pilpel

Department of Molecular Genetics, Weizmann Institute of Science, Rehovot, Israel

Description: *S. cerevisiae* cells were transformed with a Ty element under the regulation of Gal promoter. Cells were grown in media containing Galactose to induce Ty activity for ~100 generations in 15˚C. Cells were then transferred to YPD media for another 20 generations.

Rationale: The Ty element could enhance evolution in two ways. First, it might serve as a mutagen that inserts itself into new locations and introduce mutations. Second it might reverse transcribe cold responsive genes and add additional copies of them, potentially with beneficial transcription errors as DNA mutations, back into the genome.

Materials and Methods:

*S. cereivisae* cells were transformed with a Ty element under the regulation of Gal promoter into the Lys2 locus. The Ty element was amplified from a plasmid containing the Ty element under the Gal promoter (from Jef Boeke’s lab^[[1]](#footnote-1)^). The Ty was amplified using primers that contain a 50bp homology to Lys2 locus (underline) and 20bp targeting the Ty (F: GGCATCGCACAGTTTTAGCGAGGAAAACTCTTCAATAGTTTTGCCAGCGGCGGATTAGAAGCCGCCGAG, R:AATTCATATTTAATTATTGTACATGGACATATCATACGTAATGCTCAACCCCGAATACCGCAAGCGACAG). Transformation was done according to regular protocol^[[2]](#footnote-2)^.

Cells were grown in 1.2ml of YP-Gal (10g/L yeast extract, 20g/L peptone, 2% Galactose) media in a 24-well plate under constant shaking of ~800rpm at 15˚C. Upon reaching stationary phase (~48 hours) cells were diluted 1: 120 into fresh YP-Gal media. This procedure was repeated for 45 days (~100 generations). After 100 generations, cells were continued to evolve in YPD media (10g/L yeast extract, 20g/L peptone, 20g/L glucose) for another 20 generations to adapt for relevant media.

At the end of the evolution, samples from each of the evolving populations were plated on YPD-agar plates. Single colonies were picked randomly for growth experiment in a robotic system. Each colony was incubated in liquid YPD media and grown for 48-hours at 15˚C while shaking at ~800rpm, OD600 was measured at 30min intervals, and the best performing colony was chosen as the participant strains for Evolthon Challenge.

#
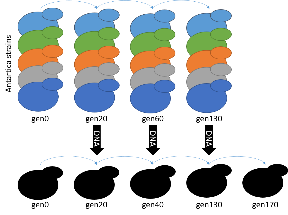
Strategy #28: Antarticold

Sivan Kaminski Strauss, Orna Dahan, Yitzhak Pilpel

Department of Molecular Genetics, Weizmann Institute of Science, Rehovot, Israel

Description: *S. cerevisiae* cells were adapted to growth in a constant temperature stress of 15˚C by daily diluting the evolving *S. cerevisiae* population. At three different time points during the evolution, the evolving cells were transformed with genomic DNA from cold-resistant strains (i.e. strains isolated from Antartica that were further evolved on 15˚C).

Rationale: Yeast species isolated from Antartica must be cold resistant. We hypothesized that they could contribute cold- resistance genes, or cold resistance version of genes.

Materials and Methods:

5 yeast species isolated from Antarctica (*Cryptococcus socialis* (YCD51), *Leucosporidium antarcticum* (YCD53), *Cryptococcus saitoi* (YCD54), *Cryptococcus vishnaicii* (YCD55), *Leucosporidium antarcticum* (YCD57)) were kindly provided Michael Springer at Harvard medical school. The 5 species were grown 1.2ml of YPD media (10g/L yeast extract, 20g/L peptone, 20g/L glucose) in a 24-well plate under constant shaking of ~800rpm at 15˚C. Upon reaching stationary phase (~48 hours) cells were diluting 1:120 into fresh YPD media. At generations 21, 63 and 130 of the evolution genomic DNA was extracted from the Antarctica yeasts, and digested with EcoRI at 37˚C, overnight.

*S. cerevisiae* cells from the organizers were grown in 1.2ml of YPD media (10g/L yeast extract, 20g/L peptone, 20g/L Glucose) in a 24-well plate under constant shaking of ~800rpm at 15˚C. Upon reaching stationary phase (~48 hours) cells were diluting 1:120 into fresh YPD media. At generations 21, 42 and 130 strains were transformed with the digested DNA from the Antarctica’s strains (from generation 21, 63, 130, respectively). After each transformation, the transformed cells were plated on YPD-agar plates, and incubated in either 4˚C or 15˚C for couple of days. Following incubation cells were collected from plates were then inoculated into 1.2ml YPD and continue evolving. Evolution was stopped after 160 generations.

At the end of the evolution, samples from each of the evolving populations were plated on YPD-agar plates. Single colonies were picked randomly for growth experiment in a robotic system. Each colony was incubated in liquid YPD media and grown for 48-hours at 15˚C while shaking at ~800rpm, OD600 was measured at 30min intervals, and the best performing colony was chosen as the participant strains for Evolthon Challenge.

#
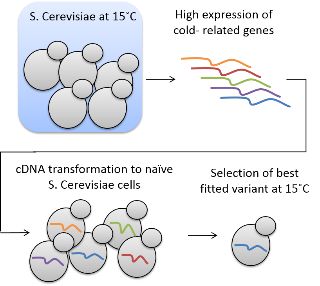
Strategy #29: Catching cold RNA

Noa Aharon Hefetz, Roni Rak, Yitzhak Pilpel

Department of Molecular Genetics, Weizmann Institute of Science, Rehovot, Israel

Description:

The transcriptome of yeast cells experiencing a cold shock (15˚C for one week) was reverse transcribed into cDNA. Naïve *S. cerevisiae* cells were transformed with the cDNA. The cDNA was designed to integrate at specific location in the genome, by homologous recombination, under the control of a strong promoter. Following transformation, to allow selection and evolution of the transformed cells, the cells were grown in a constant temperature of 15 ˚C for ~20 generations.

Rationale: We assumed that the transcriptome of cold stressed cells should over-represent cold-sensitive genes. cDNA derived from these RNAs could supplement the genome of the transformed cells with additional copies of cold-resistance genes that might improve cold resistance.

Materials and methods:

*S. cerevisiae* cells were grown in 1.2ml of YPD media in a 24-well plate under constant shaking of ~800rpm at 15˚C. Upon reaching stationary phase (~48 hours) cells were diluting 1: 120 into fresh YPD media. This procedure was repeated for one week. After one week the cell were collected and total RNA was extracted using MasterPure™ Yeast RNA Purification Kit (epicenter). cDNA was generated using “High-Capacity cDNA Reverse Transcription Kit” (Applied Biosystems), according to standard protocols. The promoter used for cDNA generation contained an polyT and homology to the *CAN1* locus 3’ end (CAN1_Rev). cDNA was cleaned using Dynabeads™ MyOne Silane beads (life technologies).

*TEF1* promoter was amplified from *S. cerevisiae* genomic DNA using a forward primer containing homology to the *CAN1* locus 5’ end and to *TEF1* promoter (CAN1_TEF1_Fwr) and phosphorylated reverse primer (TEF1_Rev). PCR product was clean using Wizard® SV Gel and PCR Clean-Up System (Promega). The PCR product, containing the *TEF1* promoter was ligated to the cDNA produced from the donor cells using T4 ligase (NEB).

*S. cerevisiae* cells from the organizers were transformed with the ligation product. The transformed cells were plated on YPD-agar plates containing canavinine (50 mg/l). All canavanine resistant colonies were pooled together and grown in the presence of canavanine (50 mg/l) for ~20 generation at 15˚C, by serial dilutions similar to the described above. At the end of the selection, samples from the evolving populations was plated on YPD-agar plates, and were incubated at 30˚C. Single colony was chosen randomly as the participant strains for Evolthon Challenge.

Primer sequences:

CAN1_Rev: 5’ GCGTGGAAATGTGATCAAAGGTAATAAAACGTCATATCTATGCTtttttttttttttttttt 3’

CAN1_TEF1_Fwr: 5’ TCAATGAAAATTTCGAGGAAGACGATAAGGTTAAGATAAGgacatggaggcccagaatacc 3’

TEF1_Rev : 5’ [Phos]ggttgtttatgttcggatgtgatg 3’

#
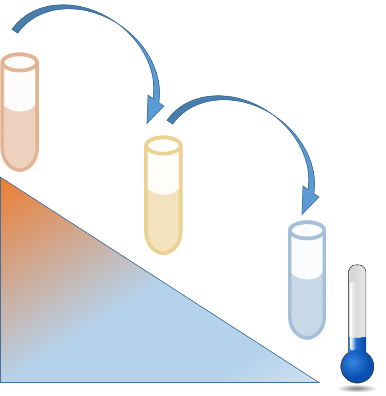
Strategy #30: *S. cerevisiae* temperature gradient

Dvir Schirman, Orna Dahan, Yitzhak Pilpel

Department of Molecular Genetics, Weizmann Institute of Science, Rehovot, Israel

Description: *S. cerevisiae* cells were adapted to growth in gradually decreasing temperature from 30˚C to 12˚C, and then gradually increase to 15˚C by daily diluting the evolving *S. cerevisiae* population.

Rational: gradual exposure to a stress has been shown in the past to allow adaptations of less costly solutions^1^.

Materials and Methods:

*S. cerevisiae* cells were grown 1.2ml of YPD media in a 24-well plate under constant shaking of ~800rpm. Upon reaching stationary phase (~48 hours) cells were diluted 1:120 into fresh YPD media. Every 14 generations (2 dilution cycles) the temperature was decreased by 1˚C. The starting temperature was 30˚c, it was decreased down to 12˚C, and then increased in the same manner up to 15˚C. The total evolution time was ~300 generations. Evolution was done in 2 repeats.

At the end of the evolution, samples from each of the evolving populations were plated on YPD-agar plates. Single colonies were picked randomly for growth experiment in a robotic system. Each colony was incubated in liquid YPD media and grown for 48-hours at 15˚C while shaking at ~800rpm, OD600 was measured at 30min intervals, and the best performing colony was chosen as the participant strains for Evolthon Challenge.

^1^Yona AH, Manor YS, Herbst RH, Romano GH, Mitchell A, Kupiec M, Pilpel Y, Dahan O. Chromosomal duplication is a transient evolutionary solution to stress. Proc Natl Acad Sci U S A. 2012 Dec 18;109(51):21010-5.

1. David J Garfinkel and others, ‘Transposon Tagging Using Ty Elements in Yeast’, 1988. [↑](#footnote-ref-1)
2. R Daniel Gietz and Robin A Woods, ‘Transformation of Yeast by Lithium Acetate/single-Stranded Carrier DNA/polyethylene Glycol Method.’, *Methods in Enzymology*, 350 (2002), 87–96 <http://www.ncbi.nlm.nih.gov/pubmed/12073338> [accessed 19 July 2018]. [↑](#footnote-ref-2)
