# Supplementary material for: Evolthon: A community endeavor to evolve lab evolution
Source: PLoS Biol. 2019 Mar 29;17(3):e3000182. doi: 10.1371/journal.pbio.3000182 (PMC6440615; doi:10.1371/journal.pbio.3000182)
Supplement: S3 Text — This file contains all materials and methods for the paper that were done in the organizer’s lab. (DOCX) [file pbio.3000182.s008.docx]

# materials and methods

## Strains and growth conditions

### Yeast

Yeast strains were based on *Saccharomyces cerevisiae* BY4741 strain. A 20-bp barcode marked with Hygromycin B resistance gene (HygR) was introduced into the HO locus to create a collection of 35 strains, each with a unique barcode (*MATa, his3Δ1, leu2Δ0, met15Δ0, ura3Δ0, HO::barcode-HygR)*.

Plasmid pAG32^1^ (Addgene plasmid #35122) was used as a template to amplify the HygR resistance cassette. The HygR was amplified with primers that include 40 bp homology to the HO locus, 20bp uniqe barcod and homology to the HygR cassata. The following primers were used

F:CTCATAAGCAGCAATCAATTCTATCTATACTTTAAAATGCTTTCTGNNNNNNNNNNNNNNNNNNNNccttgacagtcttgacgt

R: CTCAAGATACAAAAAGCGTTACCGGCACTGATTTGTTTCAACCAGggcgttagtatcgaatcg

(underlined regions are the HO homology tails, N are the 20-bp barcode, and the lower case letters are the primers for the HygR amplification from pAG32).

To introduce the barcode and the HygR cassette into the HO locus, yeast cells BY4741 were transformed with a standard LiAc protocol^2^. In short, cells in logarithmic phase were harvested and washed twice in 1M TE+LiAc. Cells were incubated with 1M TE+LiAc, 40% PEG4000, 100mg/ml salmon sperm and 45ul of PCR product for 40 minutes in 30˚C. Cells were then incubated for 40 minutes at 42˚C and incubated over-night in YPD at 30˚C. On the following day cells were plated on YPD + Hygromycin B and grown until colonies appeared to select for transformants. Insertion of barcodes was verify by amplification of the HO region using the following primers followed by Sanger sequencing. (F: ATTGTATTCAATTCCTATTC, R: ATTGTATTCAATTCCTATTC)

Unless mentioned otherwise yeast cells were grown on YPD (10g/L yeast extract, 20g/L peptone, 20g/L glucose). YPD-hyg media is YPD with 300ug/ml Hygromycin B (Roche). YPD-Sor is YPD with 1.2M of sorbitol.

### Bacteria

*E. coli* strains were based on MG1655 (K-12 F^–^ λ^–^ *ilvG*^–^ *rfb-50* *rph-1*). We have introduced a 20-bp barcode marked with Kanamycin resistance gene (KanR) into the *LacZ* locus.

KanR was amplified from a strain with genomic KanR cassette^3^ (kindly given from Ron Milo’s lab). The KanR was amplified with primers that include 40 bp homology to the *LacZ* locus, 20bp uniqe barcod and homology to the KanR cassata. The following primers were used

F:AGCGGTGCCGGAAAGCTGGCTGGAGTGCGATCTTCCTGAGGCCGATACTGNNNNNNNNNNNNNNNNNNNNggaacccctatttgttt

R:CCGCTTGCCAGCGGCTTACCATCCAGCGCCACCATCCAGTGCAGGAGCTCggaccgaaccccgcgttta

(underlined regions are the LacZ homology region, N represents the 20-bp barcode, and lower case letters are the primers for the KanR cassette)

Integration of barcodes in to the bacterial genome was based on homologous recombination using the lambda Red recombinase system^4^ using electroporation. Briefly, *E. coli* MG1655 cells harboring the pSLTS plasmid were grown over-night on LB-ampicillin media at 30˚C. 1ml of the culture was inoculated into 100ml of LB-Amp media and grown for an hour in 30˚C. L-Arabinose was added to a final concentration of 1mM and cells were allowed to grow until reaching an OD of 0.7. Cells were harvested by centrifugation (4500g for 10min) and washed twice with ice-cold 10% glycerol. Pellet was resuspended in ice-cold 10% glycerol and dispensed into 50ul aliquots.

To introduce the KanR cassette, 50-100ng of PCR products containing the cassette, barcodes and selectable marker were incubated on ice with 50ul of electrocompenet cells carrying the pSLTS plasmid. Cells were electroporated, and immediately suspended in1ml LB and incubated for 3 hours at 30˚C. Cells were then plated on LB-Kanamycin plates and grown until colonies appear to select for transformants cells. Insertion of barcodes was verify by amplification of the *LacZ* region using the following primers followed by Sanger sequencing. (F: ATGACCATGATTACGGATT, R: TTATTTTTGACACCAGACCA).

Unless mentioned otherwise *E. coli* cells were grown in LB (5g/L yeast extract, 10g/L tryptone, 10g/L NaCl). LB-Amp is LB with 100 µg/mL final concentration of Ampicilin. LB-Kan is LB with 50 µg/mL final concentration of Kanamycin. LB-NaCl is LB with 0.8M final concentration of NaCl.

## Fitness assessment using individual growth experiments

Strains were inoculated from plates containing Hygromycin B or Kanamycin (*S. cerevisiae* or *E. coli* respectively) into YPD or LB (*S. cerevisiae* or *E. coli* respectively) and grown for two days in cold temperature (*S. cerevisiae* in 15˚C, *E. coli* in 20˚C) until reaching stationary phase.

Strains were diluted 1:50 in 96-well plate for a final volume of 150ul per well (four strains in 8 repetition each and the ancestor in 48 repetitions in a single plate, in a checker-board format. Strains were grown under shaking conditions for ~50h at the appropriate temperature (*S. cerevisiae* in 15˚C, *E. coli* in 20˚C). OD600 was measured every 2 hours for ~50 hours by a plate reader (infinite 200, Tecan). All measurements were done automatically using a Hamilton robotic system.

Growth parameters (lag phase duration, growth rate at exponential phase and yield) were extracted from the obtained growth curves using the “curveball” software^5^

## Pooled competition

Strains were inoculated from plates containing Hygromycin B or Kanamycin (*S. cerevisiae* or *E. coli* respectively) into YPD or LB (*S. cerevisiae* or *E. coli* respectively) and grown for two days in cold temperature (*S. cerevisiae* in 15˚C, *E. coli* in 20˚C).

After two days, OD values were measured to each strain and strains were mixed accordingly to reach equal cell representation. The mixture was diluted 1:120 into relevant media (Yeast were competed on the following conditions: (i) YPD-Hyg at 15˚c, (II) YPD-hyg at 30˚c, (III) YPD-hyg at 8˚c and (IV) YPD-Sorbitol at 15˚c. *E.* coli were competed on the following conditions: (I) LB-kan at 20˚c, (II) LB-Kan at 37˚c, (III) LB-Kan at 8˚c and (IV) LB-NaCl) to start the competition. Cells were grown in 1.2ml liquid media under shaking of 800RPM, in a 24-well plate. Every ~1-2 days, when culture reached stationary phase, cells were diluted by factor of 1:120 and re-grown under the same conditions for 40-80 generations. Cells were frozen in 30% Glycerol and kept in -80˚c every 4 dilutions. All completions were done in 5 replicates.

## Barcode sequencing for pooled competition

At the end of the competition (~60 generations) DNA was extracted from 2, 3 or 4 time points from three repetitions (yeast: MasterPure Yeast DNA Purification Kit by epicenter, *E. coli*: Wizard Genomic DNA Purification Kit by promega). Libraries for sequencing the barcode region were constructed by designing PCR primers targeting the barcode region with tails that match Illumina adapters (yeast: F: ACGACGCTCTTCCGATCTacgtcaagactgtcaagg, R: AGACGTGTGCTCTTCCGATCTttgtattcaattcctattctaaatggc, *E. coli*: F: ACGACGCTCTTCCGATCTaaaaccctggcgttaccc, R: AGACGTGTGCTCTTCCGATCTgatccttggcggcaag. Capital letters corresponds to Illumina adaptors, while lower case letter corresponds to homology to the genome). A second PCR (F: AATGATACGGCGACCACCGAGATCTACACTCTTTCC CTACACGACGCTCTTCCGATCT, R: CAAGCAGAAGACGGCATACGAGAT***NNNNNNNN***GTGACTGGAGTTCAGACGTGTGCTCTTCCGATCT. ***N*** corresponds to Illumina index for library multiplexing) was carried out to attach the adapters for the Illumina run. Barcodes were sequenced using 75-nt single-end reads, on the NextSeq platform (Illumina).

## Pooled competition prediction based on individual growth curves

The pooled competition outcome was predicted from the individual growth curves using an expanded version of the logistic equation (**Equation 1**). The expanded version is shown in **equation 2**.

$$\boldsymbol{Eq.1} \frac{dN}{dt}=r*N*\left( 1-\frac{N}{K} \right)$$

$$\boldsymbol{Eq. 2}\frac{{dN}_{i}}{dt}=r_{i}*N_{i}*\left( 1-\frac{\alpha\sum N}{K_{i}} \right)$$

Where $r_{i}$ is the growth rate, $N_{i}$is the number of cells, $K_{i}$ is the carrying capacity of strain *i and*$\alpha$ is the effect of other strains on strain *i*. $\alpha$ was neglected, since we assumed no direct interaction between the strains. Assuming the frequency of each strain at the beginning of the competition (in this case, equal frequencies), and assigning *r* and *k* based on the individual growth experiments, the model was solved numerically to find the frequency of each strain in each time point.

## Fitness estimation based on pooled competition

Fitness was derived by employing a Maximum-Likelihood (ML) algorithm on all frequency measurements along the competition experiment per variant (fitness was calculated only for strains with more than 10 reads in the beginning of the competition). Briefly, first, each variant fitness is estimated by using a simple loglinear regression over the first three time points. Based on these estimations, the initial relative frequencies of each variant, and a noise model that accounts for experimental errors^6^, expected trajectory of each variant is estimated and compared to the measured trajectory. Next, small changes are made to our fitness estimates, comparison is repeated, fitness is updated if they better fit the data (higher likelihood). This procedure is performed iteratively until fitness estimates are stable (maximized likelihood).

1. Goldstein, A. L. & McCusker, J. H. Three new dominant drug resistance cassettes for gene disruption inSaccharomyces cerevisiae. *Yeast* **15,** 1541–1553 (1999).

2. Gietz, R. D. & Woods, R. A. Transformation of yeast by lithium acetate/single-stranded carrier DNA/polyethylene glycol method. *Methods Enzymol.* **350,** 87–96 (2002).

3. Baba, T. *et al.* Construction of Escherichia coli K-12 in-frame, single-gene knockout mutants: the Keio collection. *Mol. Syst. Biol.* **2,** 2006.0008 (2006).

4. Kim, J., Webb, A. M., Kershner, J. P., Blaskowski, S. & Copley, S. D. A versatile and highly efficient method for scarless genome editing in Escherichia coli and Salmonella enterica. *BMC Biotechnol.* **14,** 84 (2014).

5. Ram, Y. *et al.* Predicting competition results from growth curves. *bioRxiv* 22640 (2015). doi:10.1101/022640

6. Levy, S. F. *et al.* Quantitative evolutionary dynamics using high-resolution lineage tracking. *Nature* **519,** 181–186 (2015).
