# Supplementary material for: Evolthon: A community endeavor to evolve lab evolution
Source: PLoS Biol. 2019 Mar 29;17(3):e3000182. doi: 10.1371/journal.pbio.3000182 (PMC6440615; doi:10.1371/journal.pbio.3000182)
Supplement: S1 Table — (DOCX) [file pbio.3000182.s009.docx]

| **#** | **Participant name** | **Institute** | **Strategy name** | **Short description of the strategy** | **logo** |
| --- | --- | --- | --- | --- | --- |
| 1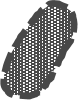 | Omri Adini, Klutstein lab and Hazan Lab | Hebrew University, Israel | Growth advantage in stationary phase | *E. coli* cells were adapted to growth in a constant temperature stress of 20˚c for two months, without any transfer or addition of nutrients. | 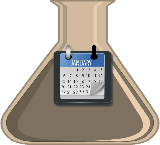 |
| 2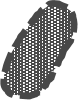 | Avihu H. Yona,  Gore lab | MIT, USA | *E. coli* Manual chemostat | To shorten lag phase and to increase growth rate, cells were diluted every morning, followed by another dilution, a couple of hours later. Experiments were performed at 15˚C | 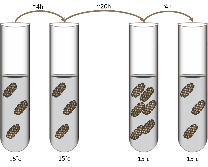 |
| 3, 9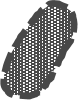 | Idan Yelin,  Kishony lab | Technion, Israel | Saltation-selection and vice versa | Strains were transformed with a cold-adapted chaperonin either before or after batch-dilution transfers for 200 generations. | 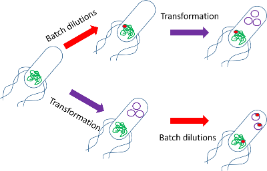 |
| 4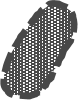 | Nilima Walunjkar,  Agashe lab | National Centre for Biological Sciences,  India | Pop-Gen | Multiple replicates of the *E. coli* strain were grown in a 48 well plate at 20˚C and diluted every 24 hours. Competition between the replicates was carried out after every 7 dilutions. | 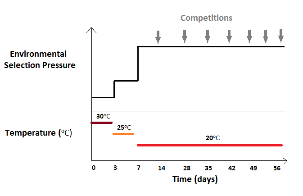 |
| 5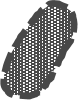 | Sivan Kaminski Strauss,  Pilpel lab | Weizmann Institute, Israel | *E. coli* Daily dilution | Cells were grown in LB in 20˚C, and were allowed to grow until stationary phase. Cells were then diluted 1:120 and re-grown in LB, at 20˚C. | 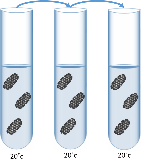 |
| 6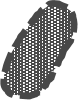 | Tanita Wein,  Dagan lab | Kiel University, Germany | Survival of the fittest by means of directional selection | The ancestral population was sampled into five subpopulations from which the fittest subpopulation was selected for the next round. The fitness was measured by a growth advantage, which is defined by fastest growth in the exponential growth phase of the populations. This approach utilizes directed selection while increasing the number of tested genotypes. The entire procedure was repeated for 100 times | 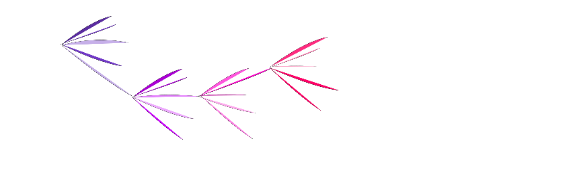 |
| 7, 8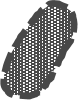 | David Wernick, Yinon Bar-On,  Milo lab | Weizmann Institute, Israel | Variable mutation-rate selection | Cells were transformed with an inducible mutator plasmid. Mutation rate was differentially induced in different repeats and cells were continuously selected for growth at 20C. A cold shock (4˚C for 1 hour) was done on strain #8 immediately after dilution | 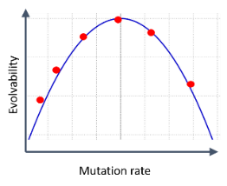 |
| 10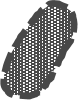 | Atray Dixit,  Regev lab | Broad Institute,  USA | Lazy man | Cells were grown at room temperature in 200 mL, diluting 1:1000 every 1-4 days | 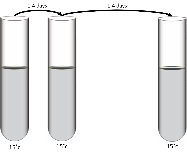 |
| 11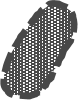 | Tim Wannier, Aditya Kunjapur,  Church lab | Harvard Medical School,  USA | Accelerated Evolution | The DNA repair gene, mutS, was disabled via Recombineering. Cells were passaged for close to two months at 1-2 passages per day after mutS was disabled, allowing an accelerated evolutionary path. | 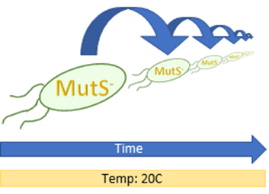 |
| 12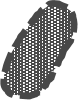 | Andreas Mershin lab | MIT,  USA | Strength through diversity: the United States *of E.coli* (U.S.E) | Ancestral monoculture was divided into over 500 subcultures, and subjected each to different "accelerated life histories" (each receiving combinations of stressors, mutagens and nutrients all grown at 18˚C). Internal competition, with the overall stress added to the propensity for horizontal gene transfer. Re-combining the divergent subcultures becomes a “United States of E. Coli” (U.S.E.) multiculture. | 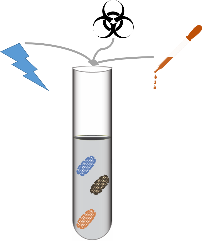 |
| 13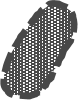 | Ghil Jona, Dikla Levi | Weizmann Institute,  Israel | Combined chemostat with temperature fluctuation | Cells were grown continuously in a chemostat at quasi-exponential phase at 20˚C. Once a week the culture was subjected to two consecutive cycles of relaxing and cold shock cycles (each of 1.5-2 hours at 30˚C followed by 15 min at 15˚C) | 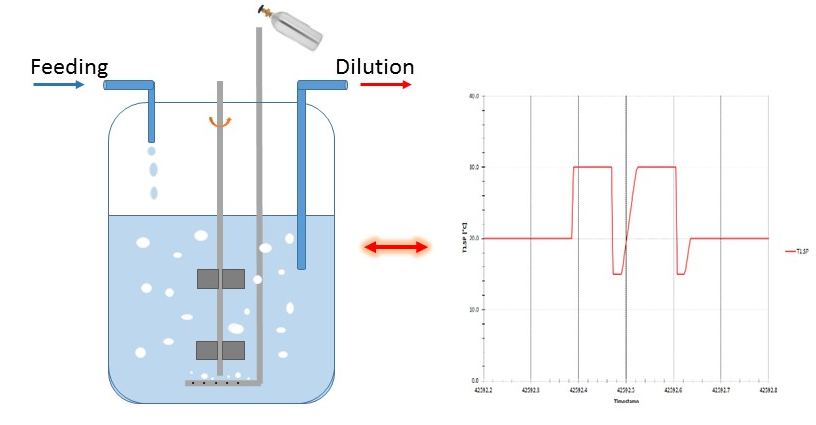 |
| 14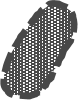 | Bálint Csörgő, Pál lab | Hungarian Academy of Sciences,  Hyngary | Hypermutation evolution | The strategy was based on the rapid capability of mutator bacteria in adapting to new environments. Cells were transformed with a mutD5 mutator allele of dnaQ, which has been shown to have a mutation rate 1000-times greater than wild-type’s level. Standard laboratory evolution techniques were executed to evolve cells for growth at 20°C in two steps, 170 generations in 25°C, followed by 170 generations in 20°C. | 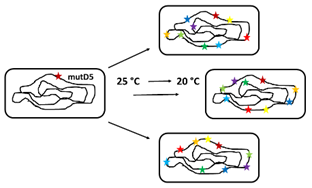 |
| 15 | Dmitry Zabezhinsky,  Gerst lab | Weizmann Institute,  Israel | Delete and prosper | Two genes that were shown to confer cold sensitivity were knocked out (OSH3 and TOR1) and two genes, CTO1 and PHO90, that confers cold resistance were over expressed by fusion to the strong TDH3 promoter | 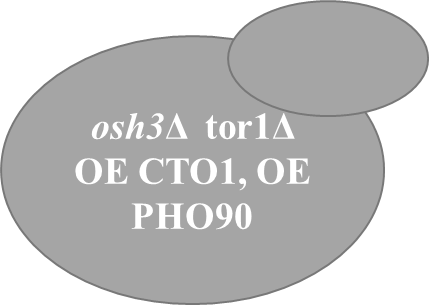 |
| 16 | Joseph Georgeson, Ehud Sass,  Levy lab | Weizmann Institute,  Israel | Chemical mutagenesis | Cells were exposed to EMS mutagenesis. Rapid selection was achieved through pooling fastest growing colonies. Work was assisted by high-throughput robot | 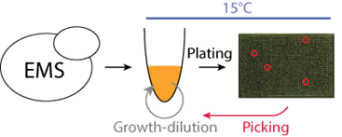 |
| 17 | Alex N. Nguyen Ba,  Desai lab | Harvard University,  USA | Breeding with natural variation | Cells were cycled between rounds of outbreeding and rounds of selection. To accelerate the breeding process, the selection temperature was continuously decreased over breeding cycles. | 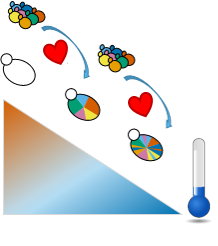 |
| 18 | Cambridge Biochemistry student's team,  Ralser lab | University of Cambridge,  U.K. | Simply Metabolism | Metabolic deficiencies of the ancestor have been repaired, then the strain was rendered diploid as most metabolic evolution did occur upon genome duplication. Then, the metabolically competent diploid was evolved for fast growth at low temperature. | 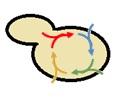 |
| 19 | Razi Zeidan,  Nachman lab | Tel Aviv University,  Israel | Adaptive lab evolution with mating | Different wild strains were competed to detect the strain most resistant to cold temperatures. Winning strain was crossed with original strain, followed by more back-crossed procedures. After several rounds, cells were sporulated and tested in 15˚C. Best performing strain on 15˚C was chosen. | 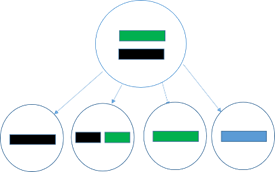 |
| 20 | Avihu H. Yona,  Gore lab | MIT,  USA | *S. cerevisiae* Manual chemostat | To shorten lag phase and increase growth rate, cells were diluted every morning, followed by another dilution a couple of hours later. By this, cells were selected to rush the exit from stationary phase and to have high growth rate in early log phase. | 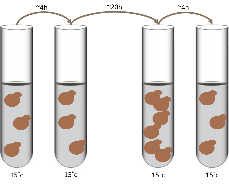 |
| 21 | Shira Nouriel,  Klutsteinlab and Hazan Lab | Hebrew University,  Israel | Foodie-evolution | Repeated transformation of genomic DNA from cold-resistant yeast and growth in cold conditions | 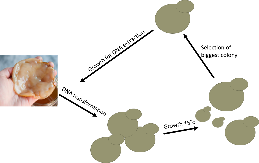 |
| 22 | Sivan Kaminski Strauss,  Pilpel lab | Weizmann Institute,  Israel | *S. cerevisiae* Daily dilution | Cells were grown in YPD in 15˚C, and allowed to grow until stationary phase. Cells were then diluted 1:120 and re-grown in LB, 15˚C. | 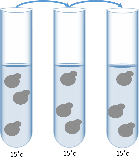 |
| 23 | Ghil Jona, Dikla Levi | Weizmann Institute,  Israel | Combined chemostat with temperature fluctuation | Cells were grown continuously in a chemostat at quasi-exponential phase at 20˚C. Once a week the culture was subjected to two consecutive cycles of relaxing and cold shock cycles (each of 1.5-2 hours at 30˚C followed by 15 min at 15˚C) | 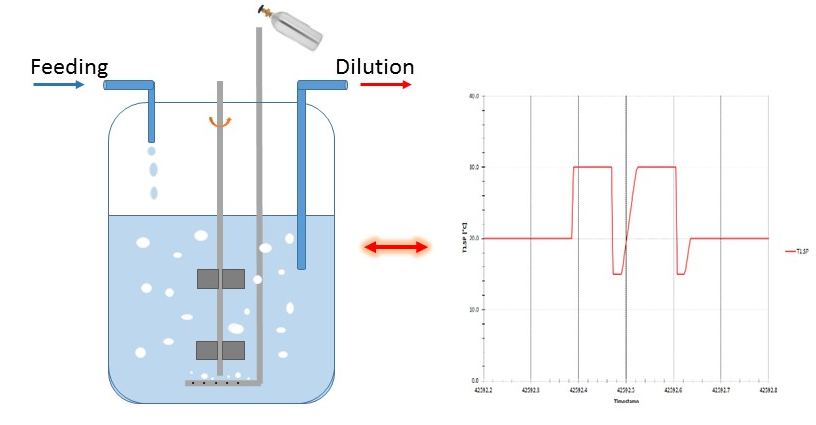 |
| 24 | Steinmetz_team, Stienmetz lab | EMBL,  Germany | Engineering of cold response genes using CRISPR/Cas9 | Cells were engineered using CRISPR/Cas9 to generate loss-of-function alleles of genes conferring cold sensitivity and introduce short, highly active, synthetic promoters in front of genes whose overexpression has been linked to decreased cold sensitivity. | 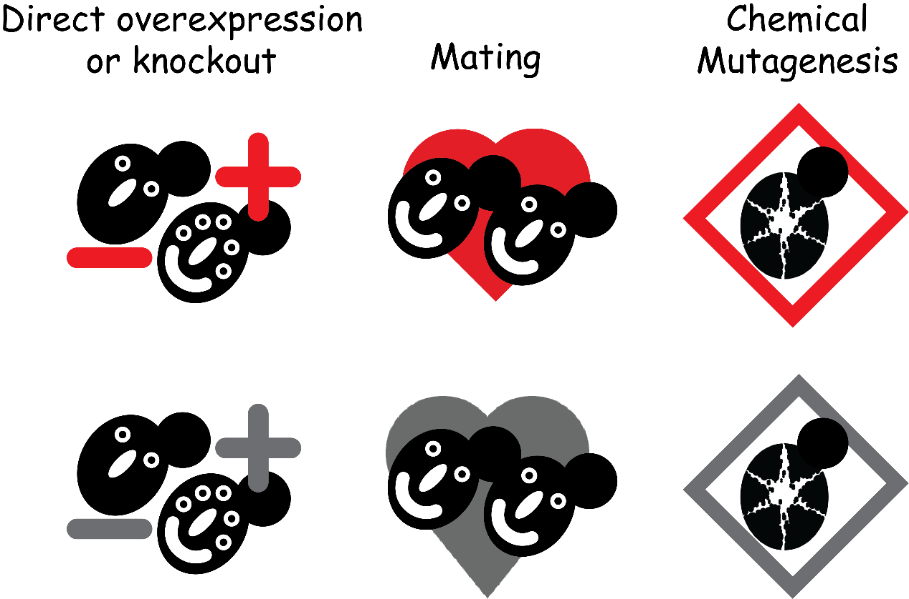 |
| 25 | Steinmetz_team, Stienmetz lab | EMBL,  Germany | Cycles of random mutagenesis with selection | Cells recieved cycles of random chemical or UV mutagenesis and selection of best performers. After each round of mutagenesis the 10% best performing strains were identified by phenotyping at 15°C and used in two separate pools (best half, other half) for the next round of mutagenesis. | 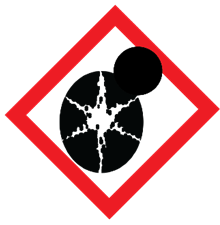 |
| 26 | Steinmetz_team, Stienmetz lab | EMBL,  Germany | Mating | Cells were with a wild type strain with superior growth performance at 15°C, followed by a round of random mating (two rounds of interbreeding total). | 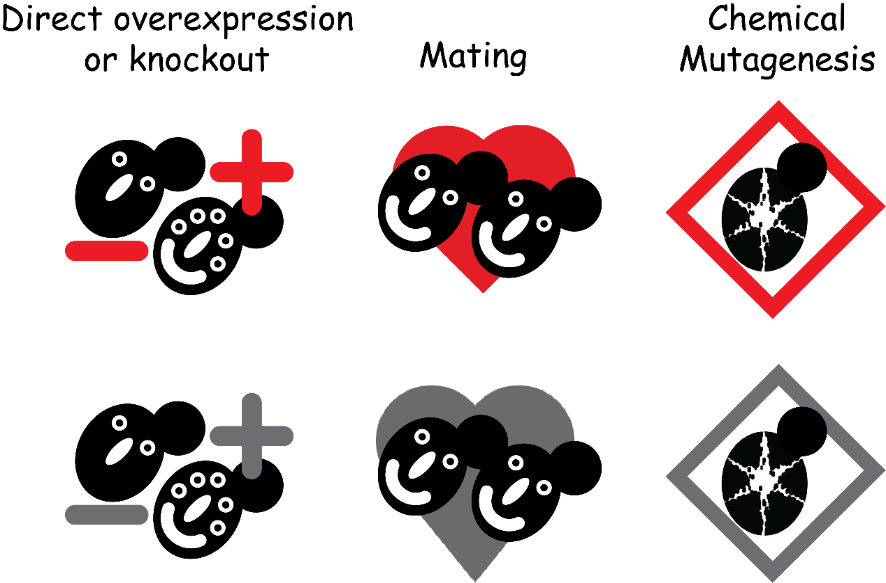 |
| 27 | Sivan Kaminski Strauss,  Pilpel lab | Weizmann Institute,  Israel | Ty-induced evolution | Cells were transformed with the Ty transposable element under the regulation of Gal promoter. Cells were grown in media containing Galactose to induce Ty activity for ~100 generations. Cells were transferred into YPD (to get use to the media) for another 20 generations. | 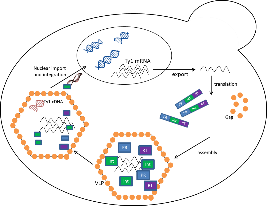 |
| 28 | Sivan Kaminski Strauss and Orna Dahan,  Pilpel lab | Weizmann Institute,  Israel | Antarticold | We used genomic DNA of Yeast strains isolated from Antarctica. Every ~50 generations DNA was extracted from Antarctica’s strains and was massively transformation into our barcoded strain. Immediately after transformation strains were incubated in 15˚C, as a selective pressure and then continued standard evolution. | 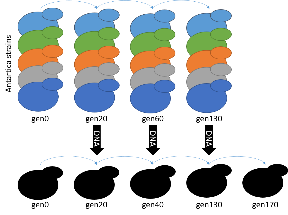 |
| 29 | Noa Hefetz and Roni Rak,  Pilpel lab | Weizmann Institute,  Israel | Catching cold RNA | Cells were grown on 15˚C for one week, RNA was extracted, reverse transcribed to create cDNA which was then transformed into CAN1 gene (by homologous recombination) to new yeast cells. Cells were evolved on canavanine media. | 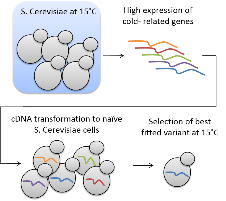 |
| 30 | Dvir Schirman,  Pilpel lab | Weizmann Institute,  Israel | *S. cerevisiae* temperature gradient | Yeast cells were evolved on gradually declining temperature. At the start of the evolution the cells were grown on 30˚C, and once every 2 dilutions the temperature was lowered by one degree. The temperature was lowered in this manner down to 12 degrees, and then risen back to 15˚C in the same gradual manner. Total time of the evolution was about 300 generations. | 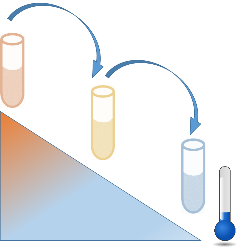 |
